# Glycolytic reprogramming and cell-specific lactylation in asthma: an evidence-graded framework for testable metabolic-epigenetic profiles

**DOI:** 10.3389/fimmu.2026.1955426

**Published:** 2026-09-15

**Authors:** Shu Li, Haoxiang Fang, Kun Wang

**Affiliations:** 1 The First Clinical Medical College of Anhui University of Chinese Medicine, Hefei, China; 2 Huixue Research Center, Anhui University of Chinese Medicine, Hefei, China; 3 College of Traditional Chinese Medicine, Anhui University of Chinese Medicine, Hefei, China

**Keywords:** asthma, chromatin regulation, glycolytic reprogramming, immunometabolism, lysine lactylation

## Abstract

Asthma is metabolically heterogeneous, but existing syntheses often place patient observations, animal perturbations, cross-disease mechanisms, and therapeutic concepts on the same evidentiary plane. This review addresses that problem by organizing glycolytic reprogramming and lysine lactylation through a three-tier hierarchy: human asthma observations, mechanistic perturbation in asthma-relevant models, and cross-disease or conceptual precedents. Mitochondrial and redox disturbances alter glycolytic and oxidative capacity across immune and structural cells, whereas lactate can function separately as an exported acidifying metabolite and as an intracellular substrate for site-specific lactylation. Four asthma-associated circuits have the strongest direct mechanistic support: epithelial histone H3 lysine 18 lactylation (H3K18la)–colony-stimulating factor 1 receptor (CSF1R) signaling; cluster of differentiation 4-positive (CD4^+^) T-cell H3K18la–dipeptidyl peptidase 4 (DPP4) regulation; mitochondrial phosphoenolpyruvate carboxykinase 2 (PCK2)-dependent polyglutamine-binding protein 1 (PQBP1) K223 lactylation coupled to protein arginine methyltransferase 5 (PRMT5) inhibition; and macrophage sirtuin 6 (SIRT6)–lactate dehydrogenase A (LDHA)–histone H4 lysine 12 lactylation (H4K12la) signaling. Their prevalence, effect size, temporal stability, and distribution across human asthma phenotypes remain unknown, so they should not be considered validated endotypes or biomarkers. We therefore propose five mechanistically anchored research profiles and sequential analytical, biological, clinical, and external-replication gates, with falsification criteria and no universal numerical cutoffs absent empirical calibration. We further distinguish airway-restricted delivery from regional and cellular selectivity and define deposition, diseased-mucus transport, cell-resolved pharmacokinetics and pharmacodynamics, intracellular target engagement, and local-to-systemic safety as translational gates. The distinctive contribution is an evidence-graded, falsifiable framework that links metabolic state to cell- and residue-specific lactylation while making explicit what remains to be demonstrated in human asthma.

## 1 Introduction

Asthma is a heterogeneous chronic airway disease characterized by variable inflammation, airflow limitation, and remodeling. Type 2 pathways involving T helper 2 (Th2) cells, group 2 innate lymphoid cells (ILC2s), immunoglobulin E (IgE), and their effector cytokines dominate many phenotypes but do not account for all forms of severe, mixed-granulocytic, or treatment-refractory disease (1). The efficacy of interleukin-4 receptor alpha (IL-4Rα) blockade with dupilumab illustrates the value of cytokine-directed therapy while highlighting biological processes that may persist outside the targeted pathway (2).

Immunometabolism offers one route into this residual heterogeneity because metabolic programs constrain immune-cell activation, differentiation, and effector function (3, 4). Asthma-focused reviews place glycolysis within broader networks of fatty-acid and amino-acid metabolism (5, 6). A controlled allergen-challenge model resolved epithelial glycolytic programs, pathogenic Th2 cells expressing interleukin-9 (IL-9), and asthma-specific contacts between phagocytes and basal cells (7). By contrast, circulating T cells from severe allergic asthma showed downregulation of several metabolic pathways relative to mild disease (8). The airway–blood discordance makes sampling compartment and activation state part of the biological result, not a technical footnote.

Allergens, hypoxia, pollutants, toxicants, and mechanical stress can disrupt mitochondrial function and redox balance in the airway. Oxidative and nitrosative stress have been documented across obstructive lung diseases, although their magnitude and consequences vary by disease and patient group (9). In models of severe asthma, histidine metabolism, oxidative stress, mitochondrial DNA (mtDNA) release, and epithelial necroptosis form a recently defined pathway of tissue injury (10).

These disturbances converge on metabolic regulators. T-cell-intrinsic nuclear factor erythroid 2-related factor 2 (NRF2) sustains the bioenergetic capacity of polyfunctional Th2 responses (11), whereas long- and short-form epithelial thymic stromal lymphopoietin (TSLP) exert opposing control over hypoxia-inducible factor 1 alpha (HIF-1α)-dependent glycolysis (12). Dexamethasone reduced HIF-1α signaling, glycolytic enzymes, lactate abundance, and protein lactylation in a murine model (13). The central mechanistic question is ordering: in human asthma, lactylation may drive steroid-responsive transcription, record an upstream metabolic reset, or simply accompany it.

Lactate has at least two separable roles in the asthmatic airway: monocarboxylate transporter (MCT)-coupled proton export, which can contribute to extracellular acidification, and intracellular lysine lactylation (Kla). Mechanistic interpretation therefore requires explicit identification of the lactate-producing or receiving cell, subcellular compartment, modified residue, and disease context (14). The most direct asthma evidence currently centers on four circuits: epithelial histone H3 lysine 18 lactylation (H3K18la)–colony-stimulating factor 1 receptor (CSF1R) signaling; cluster of differentiation 4-positive (CD4^+^) T-cell H3K18la–dipeptidyl peptidase 4 (DPP4) regulation; mitochondrial phosphoenolpyruvate carboxykinase 2 (PCK2)-dependent polyglutamine-binding protein 1 (PQBP1) K223 lactylation coupled to protein arginine methyltransferase 5 (PRMT5) inhibition; and macrophage sirtuin 6 (SIRT6)–lactate dehydrogenase A (LDHA)–histone H4 lysine 12 lactylation (H4K12la) signaling (15–17, 46). By contrast, studies of candidate compounds directed at the p300 histone acetyltransferase and histone deacetylase (HDAC)-related compartmental control (18, 19), bromodomain-containing protein 4 (BRD4)-dependent remodeling (20), T helper 9 (Th9) metabolic licensing (21), NLR family pyrin domain-containing 3 (NLRP3) inflammasome activity (22), gut-derived cinnamic-acid signaling (23), and mechanical compression (24) provide adjacent mechanistic or contextual evidence. These observations should not be interpreted as equivalent proof of lactylation-dependent pathways in patients with asthma.

This review therefore places direct asthma evidence at the center of the synthesis and applies a three-tier hierarchy throughout: human asthma observations; mechanistic perturbation in asthma-relevant cell and animal models; and cross-disease or conceptual precedents. It traces the pathway from mitochondrial and redox disturbance through glycolytic flux and lactate compartmentalization to four cell- and residue-specific circuits: epithelial H3K18la–CSF1R signaling, CD4^+^ T-cell H3K18la–DPP4 regulation, epithelial PCK2-dependent PQBP1 K223 lactylation coupled to PRMT5 inhibition, and macrophage SIRT6–LDHA–H4K12la signaling. These circuits are evaluated as mechanistic research constructs rather than validated clinical endotypes because their prevalence, effect size, and distribution across human asthma phenotypes have not been established. Broader immune, structural, systemic, and cross-disease findings are included only when they sharpen a testable asthma-specific question or define an inferential boundary.

## 2 Mitochondrial and redox inputs to glycolytic reprogramming

### 2.1 Environmental stress, mitochondrial quality control, and redox imbalance

Particulate and chemical stressors can reconfigure mitochondrial respiration. diisononyl phthalate (DiNP)-exposed asthmatic mice showed impaired respiratory-chain activity, oxidative imbalance, and altered apoptotic signaling in lung and neural tissues (25). Repeated low-dose exposure to fine or quasi-ultrafine particulate matter increased oxidative phosphorylation and mitochondrial mass in bronchial epithelial cells while simultaneously increasing superoxide production and mitochondrial fission, particularly after quasi-ultrafine exposure (26). Compensatory respiration and oxidative injury can therefore coexist; this paired phenotype belongs to the tested exposure systems and should not be treated as a universal human-asthma signature.

Allergen-driven epithelial metabolism is also controlled at the membrane. In bronchial epithelial models, fucosyltransferase 8 (FUT8) increased house dust mite (HDM)-induced glycolysis, epithelial–mesenchymal transition (EMT), and inflammation by modifying glucose transporter 1 (GLUT1) N-glycosylation (27). Clinical resolution of the HDM–FUT8–GLUT1 axis now depends on measuring FUT8 activity and GLUT1 glycoforms directly in patient airway epithelium.

Asthma and related obstructive lung diseases are associated with elevated levels of oxidative products, including 3-nitrotyrosine, 3-bromotyrosine, 3-chlorotyrosine, and malondialdehyde (MDA), often accompanied by lower levels or activity of reduced glutathione (GSH), catalase (CAT), or superoxide dismutase (SOD). Reduced nicotinamide adenine dinucleotide phosphate (NADPH) oxidase 4 (NOX4)-derived hydrogen peroxide is one potential pulmonary redox signal (28), and its effects depend on concentration, timing, and cellular context rather than reflecting nonspecific toxicity alone.

### 2.2 Histidine metabolism, mtDNA release, and epithelial necroptosis

In severe-asthma models, increased histamine synthesis and carnosine depletion drove oxidative stress, mitochondrial dysfunction, and mtDNA fragmentation and release. Released mtDNA engaged a route dependent on mitochondrially encoded NADH dehydrogenase 6 (mt-ND6) and formyl peptide receptor 2 (FPR2) that promoted epithelial necroptosis and airway inflammation (10). Genetic and pharmacological perturbation anchors causality within the experimental systems. Translation, however, hinges on determining how often this histidine–mtDNA circuit is active in human severe asthma and whether it marks a distinct inflammatory state.

### 2.3 Redox-sensitive control of glycolytic capacity

NRF2 regulates more than antioxidant gene expression. In allergen-responsive CD4^+^ T cells, cell-intrinsic NRF2 maintained glycolysis, oxidative phosphorylation (OXPHOS), and polyfunctional Th2 responses through a peroxisome proliferator-activated receptor gamma (PPARγ)-linked program (11). Brahma-related gene 1 (BRG1)-dependent chromatin remodeling controls ILC2 effector and memory metabolism (29), whereas melatonin increased NADPH availability and NRF2-dependent GSH accumulation while reducing ILC2-driven airway hyperresponsiveness in experimental models (30). Redox adaptation is therefore an active bioenergetic program in type 2 immunity, not merely a damage-control response.

The stimulator of interferon genes (STING)–HIF-1α axis provides another entry into glycolytic reprogramming. Qufeng Xuanbi formula suppressed this axis in HDM-induced asthma models (31), whereas formaldehyde enhanced HIF-1α-associated macrophage inflammation in mice (32). HIF-1α can induce hexokinase 2 (HK2), pyruvate kinase M2 (PKM2), and LDHA, and dimeric PKM2 may function as a nuclear transcriptional co-regulator. The pharmacology is less mature than the pathway model: the PFKFB3 inhibitor 3PO and 3,6′-disinapoyl sucrose reduced inflammatory activity through 6-phosphofructo-2-kinase/fructose-2,6-bisphosphatase 3 (PFKFB3)- or UDP-galactose 4-epimerase (GALE)-associated metabolic regulation (33, 34), but target selectivity, exposure–response relationships, and chronic-airway safety have not been defined.

Airway structural cells sustain glycolysis through additional routes. Midkine increased PKM2 and LDHA expression through phosphoinositide 3-kinase–protein kinase B (PI3K–AKT) signaling in airway smooth-muscle cells (35). In airway epithelium, HDM-induced long-form TSLP increased microRNA-223 (miR-223), suppressed von Hippel–Lindau tumor suppressor (VHL), and stabilized HIF-1α, whereas short-form TSLP opposed the same response (12). Viral exacerbations introduce a different problem: HIF-1α and ORMDL sphingolipid biosynthesis regulator 3 (ORMDL3)–ceramide signaling may force dendritic cells toward glycolysis, intensify local nutrient competition, and restrict interferon-γ production by natural killer cells. Testing this putative metabolic synapse will require paired measurements of substrate flux and cell function in patient airways (36).

### 2.4 Cell-specific entry points into epithelial and myeloid glycolysis

Human and experimental data place IL-1 upstream of epithelial glycolysis. Sputum lactate was higher in asthma, particularly in neutrophil-rich samples; it correlated positively with IL-1β and inversely with lung function. IL-1α or IL-1β increased epithelial glucose utilization, LDHA expression, lactate production, and cytokine release, whereas inhibition of the kinase IKKϵ (inhibitor of nuclear factor kappa-B kinase subunit epsilon) or of LDHA reduced disease features in mice (37). IL-1β also induced glutathionylation of metabolic proteins; loss of glutaredoxin 1 amplified glycolysis and TSLP release, both of which were reduced by glycolytic blockade (38).

Other epithelial pathways converge on glycolysis. HDM-induced macrophage migration inhibitory factor (MIF) increased glycolytic flux, cytokine production, and disruption of E-cadherin organization; MIF silencing or the PFKFB3 inhibitor PFK15 attenuated these effects (39). Epithelial HK2 was also increased in clinical and experimental samples, and its knockdown reduced inflammation and airway hyperresponsiveness through a peptidylprolyl isomerase F-dependent apoptotic mechanism rather than the canonical voltage-dependent anion channel 1 (VDAC1) interaction (40).

Pyruvate handling differs among epithelial lineages. Glycolytic activity was increased in club and goblet cells from asthma samples, but club-cell mitochondrial pyruvate carrier 2 (MPC2) deletion, unlike LDHA deletion, reduced goblet-cell differentiation and type 2 inflammation through a CXCL17–CXCR4 macrophage circuit (41). Mitochondrial pyruvate import and cytosolic lactate production are therefore not interchangeable. Formaldehyde further increased glycolysis and cytokine production primarily when allergic inflammation was combined with lipopolysaccharide (42) (**Figure 1**, **Table 1**).

**Figure 1:**
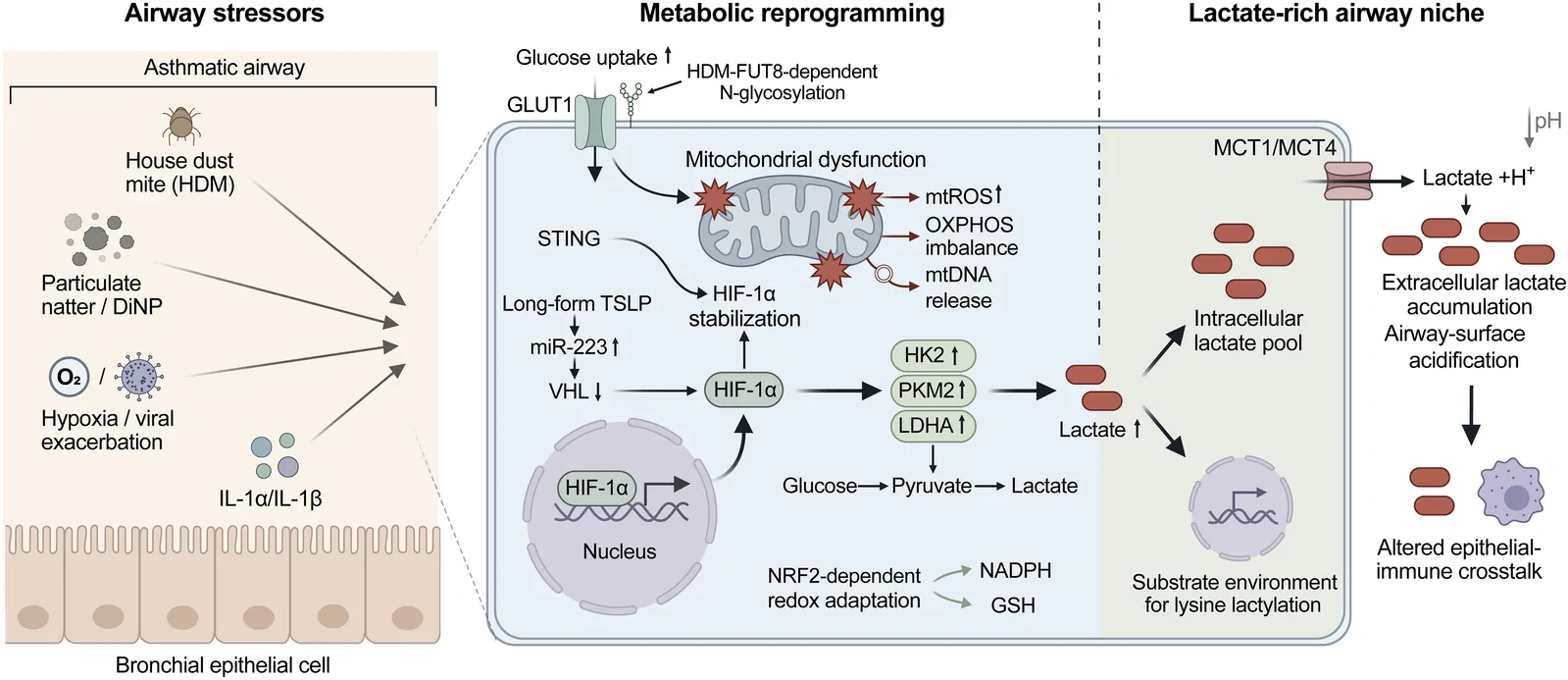
Integrated framework linking metabolic reprogramming, lactate compartmentalization, and cell-specific lactylation in asthma. Airway stressors perturb mitochondrial function, redox balance, and glycolytic flux, thereby expanding intracellular lactate pools that support epithelial H3K18la–CSF1R signaling, CD4^+^ T-cell H3K18la–DPP4 regulation, PCK2-dependent PQBP1 K223 lactylation, and macrophage SIRT6–LDHA–H4K12la signaling. MCT1/MCT4-mediated export of lactate and protons promotes extracellular lactate accumulation and airway-surface acidification. This integrative schematic combines human observations with preclinical mechanisms; pathway-specific evidence levels and remaining validation gaps are detailed in **Figures 2**–**5** and **Tables 1**, **2**, **4**.

**Table 1:** Key upstream regulators of glycolytic reprogramming in asthma.

Trigger or intervention	Principal cell context	Mechanistic axis and effect	Evidence source/model	Interpretive boundary	Ref.
Environmental particulates or toxicants	Airway epithelium and lung tissue	Respiratory-chain dysfunction, ROS, and mitochondrial fission; impaired respiration or compensatory OXPHOS	Mouse exposure models and *in vitro* cellular studies	Direct patient-airway validation and asthma generalizability are not established	(25, 26)
House dust mite	Bronchial epithelium	FUT8-dependent GLUT1 N-glycosylation; increased glucose uptake, glycolysis, inflammation, and EMT	Mechanistic bronchial-epithelial models	Frequency and effect size in human asthmatic airways have not been quantified	(27)
IL-1α or IL-1β	Airway and basal epithelium	IKKϵ–LDHA and glutathionylation–GLRX1; increased glycolysis, lactate, cytokines, and TSLP	Human asthma sputum association; primary epithelial-cell perturbation; mouse asthma models	Mixed sputum cannot assign the lactate signal to a producing cell; flux tracing is required	(37, 38)
HDM-induced MIF or epithelial HK2	Bronchial epithelium	MIF–PFKFB3 and HK2–PPIF; glycolysis, barrier disruption, apoptosis, and airway inflammation	HK2 reported in clinical and experimental samples; epithelial perturbation and asthma models	Direct protein lactylation was not tested; the human relevance of the MIF arm remains unresolved	(39, 40)
STING/HIF-1α signaling and formaldehyde	Airway immune and structural cells	STING–HIF-1α and context-dependent HIF-1α activation; increased glycolytic-enzyme expression and inflammatory signaling	Mouse and other preclinical asthma models	No direct patient-airway validation; formaldehyde effects depend on the inflammatory context	(31, 32, 42)
ILC2 redox adaptation	ILC2s	BRG1 and NRF2–NADPH–GSH; metabolic support of effector and memory-like states	Predominantly mouse and cellular models	Human airway ILC2 states require longitudinal cell-resolved validation	(29, 30)
Midkine and PDGF-BB	Airway smooth-muscle cells	PI3K–AKT–PKM2/LDHA; increased glycolysis, proliferation, and migration	Airway smooth-muscle cell culture	No patient-tissue validation or site-specific lactylation assay	(35)
Viral or hypoxic exacerbation	Dendritic-cell–NK-cell interface	ORMDL3/ceramide–HIF-1α and nutrient competition; local lactate and reduced NK-cell IFN-γ	Conceptual and indirect cellular evidence	Paired patient-airway flux and cell-function data are absent; the metabolic synapse remains putative	(36)
Mitochondrial pyruvate import	Club and goblet cells; macrophages	MPC2–CXCL17–CXCR4; regulation of goblet differentiation and type 2 inflammation independently of LDHA	Human asthma cell-state observations plus lineage-restricted mouse genetics	Human observations are associative; causal separation of MPC2 and LDHA derives from mouse genetics	(41)
Glycolytic interventions	Experimental asthma	PFKFB3 inhibition (3PO) and GALE-associated reprogramming; reduced glycolysis-related inflammation	Preclinical pharmacological and mouse-model studies	Selectivity, exposure–response, and causal linkage to lactylation remain unresolved	(33, 34)

## 3 Lactylation and chromatin regulation

### 3.1 p300 and site-selective histone acylation

In asthma, the strongest evidence linking p300 to lactylation comes from the epithelial H3K18la–CSF1R study. Clinical specimens and single-cell data showed increased epithelial glycolysis and histone lactylation, with H3K18la enriched at the CSF1R promoter; glycolytic inhibition or p300 knockdown reduced H3K18la and inflammatory output, whereas lactate supplementation increased both. Chromatin immunoprecipitation sequencing (ChIP-seq) and transcriptomic profiling resolved a colony-stimulating factor 1 (CSF1)–CSF1R–mitogen-activated protein kinase (MAPK) autocrine loop (15). These perturbations support p300 dependence within the tested system, but they do not by themselves establish p300 as the principal lactylation writer in primary asthmatic airway cells. Direct assignment will require purified catalytic assays, site-specific rescue, and intracellular target-engagement measurements in disease-relevant primary cells.

At the broader biochemical and pharmacological level, p300 has been reported to catalyze lysine lactylation (14), but the relative contribution of enzymatic writing versus nonenzymatic acyl transfer in airway cells remains unresolved. PU139 and PU141 were assessed by docking, molecular dynamics, and binding-energy calculations (18), whereas cerevisterol entered the asthma literature through network analysis and experimental work on Bailing Capsule (43). These studies prioritize possible p300 interactions; they do not demonstrate intracellular engagement or correction of asthma-associated lactylation (44). The p300-directed small molecule LTK14, cerevisterol, garcinol, and related compounds should therefore remain candidate tools rather than validated airway p300 inhibitors. Genomic studies of steroid-responsive regulators such as Krüppel-like factor 9 (KLF9) provide adjacent chromatin context (45), not evidence of p300-mediated lactylation in asthma. Population mapping across airway compartments and clinical phenotypes also remains necessary before the H3K18la–CSF1R circuit can be treated as a prevalent asthma endotype (**Figure 2**).

**Figure 2:**
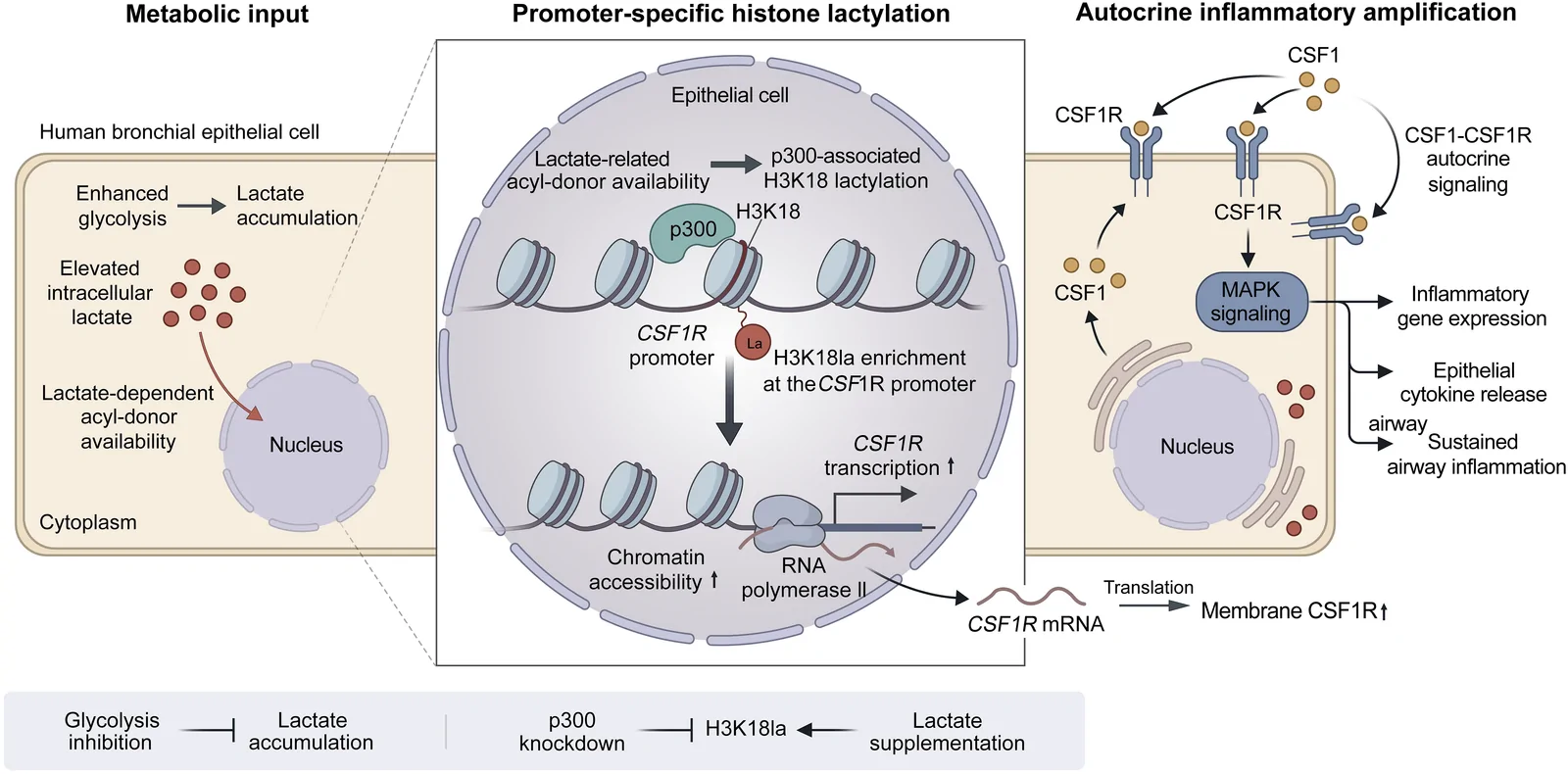
Epithelial H3K18 lactylation drives CSF1–CSF1R autocrine amplification in asthma. Glycolysis-derived lactate supports p300-associated H3K18la enrichment at the CSF1R promoter, increasing CSF1R transcription, membrane CSF1R expression, and CSF1–CSF1R–MAPK signaling. Glycolytic inhibition or p300 knockdown suppresses the circuit, whereas lactate supplementation amplifies it. Evidence is derived from human specimens, single-cell analysis, chromatin and transcriptomic profiling, and mechanistic perturbation; its distribution across airway regions and clinical endotypes requires further mapping.

A second H3K18la circuit operates in CD4^+^ T cells. In a murine asthma model, increased glycolysis enriched H3K18la at the DPP4 promoter and increased DPP4 transcription. DPP4 inhibition curtailed T helper 17 (Th17) differentiation, and co-culture linked the T-cell program to epithelial EMT (46). Mouse genetics and co-culture establish internal mechanistic coherence. Determining whether airway-infiltrating CD4^+^ T cells carry the same locus-specific mark in patients will require endobronchial sampling and cell-resolved chromatin assays (**Figure 3**).

**Figure 3:**
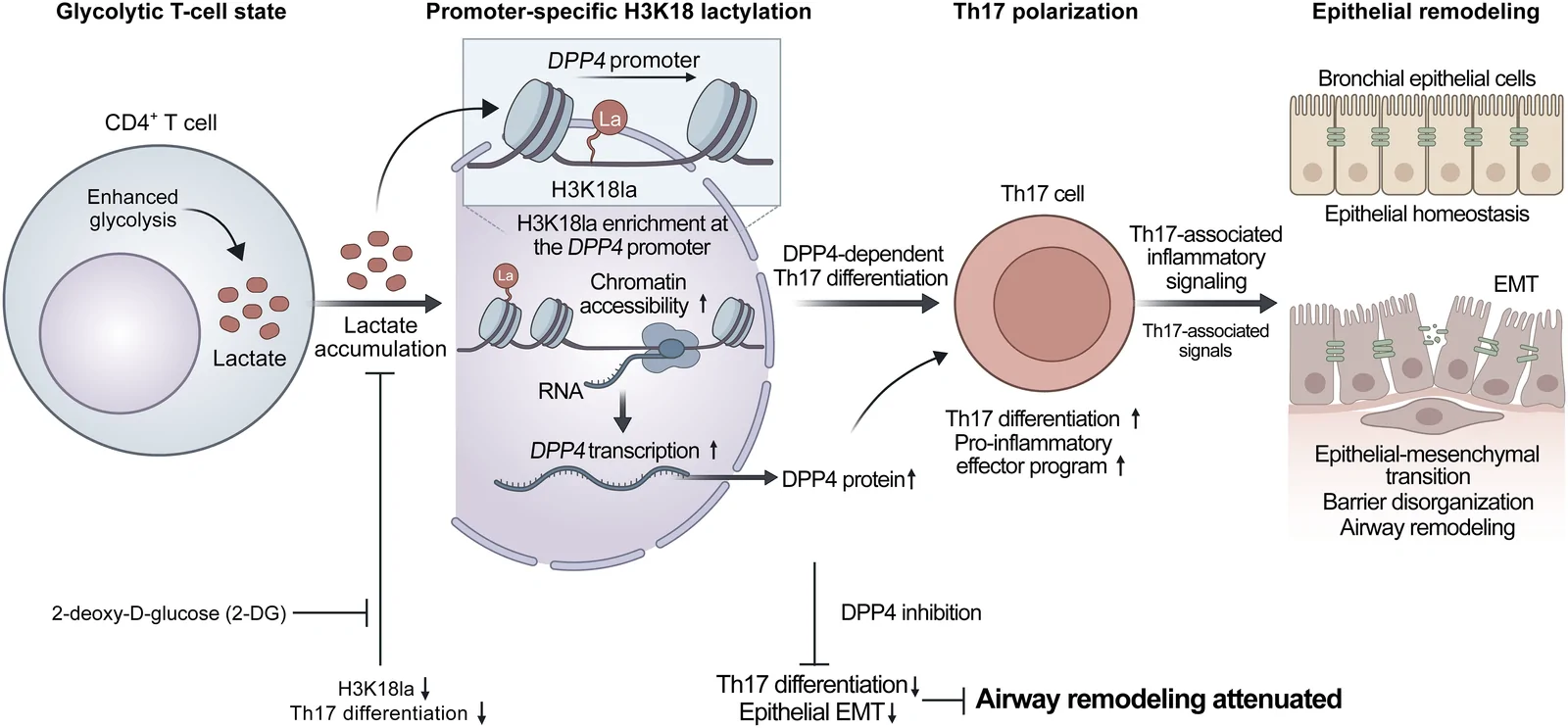
CD4^+^ T-cell H3K18 lactylation activates a DPP4–Th17 remodeling circuit. Increased T-cell glycolysis enriches H3K18la at the DPP4 promoter, increases DPP4 transcription and protein expression, and promotes Th17 differentiation. Th17-associated inflammatory signals subsequently disrupt epithelial organization and drive epithelial–mesenchymal transition and airway remodeling. Treatment with 2-deoxy-D-glucose or DPP4 inhibition attenuates the circuit in mouse and co-culture systems. Cell-resolved chromatin analysis of airway-infiltrating CD4^+^ T cells represents the key human validation step.

Two adjacent observations provide context but not asthma-lactylation evidence. In chronic obstructive pulmonary disease (COPD), LDHA-dependent histone H3 lysine 14 lactylation (H3K14la) at the low-density lipoprotein receptor-related protein 5 (LRP5) promoter increased β-catenin signaling and reduced occludin and zonula occludens-1 (ZO-1); oxamate or LDHA/LRP5 knockdown partially restored barrier markers (47). Asthmatic airway smooth-muscle cells exhibit p300- and bromodomain and extra-terminal domain (BET)-associated histone H3 acetylation at the CXCL8 promoter (48). The first study is cross-disease, and the second concerns acetylation rather than lactylation; neither establishes an equivalent epithelial H3K18la mechanism in asthma.

### 3.2 Nonhistone lactylation and coupled acylation circuits

Multiomic analysis of HDM-induced asthma resolved a coupled succinylation–lactylation circuit centered on PCK2 and PQBP1. PCK2 K100 succinylation antagonizes ubiquitination-mediated turnover, stabilizing PCK2 and expanding the intracellular lactate pool. The resulting lactate accumulation drives PQBP1 K223 lactylation, suppresses the PRMT5–WD repeat domain 77 (WDR77) methyltransferase complex, depletes repressive histone H4 arginine 3 symmetric dimethylation (H4R3me2s) at inflammatory promoters, and releases MAPK-pathway genes from transcriptional restraint (16). Conditional epithelial *Pqbp1* deletion, PCK2 knockdown, and oxamate each reduced inflammation and goblet-cell metaplasia *in vivo* (**Figure 4**; **Supplementary Figure S1**). Translating this residue-specific circuit to clinical disease now requires targeted mass-spectrometric detection of PCK2 K100 succinylation and PQBP1 K223 lactylation in human endobronchial biopsies.

**Figure 4:**
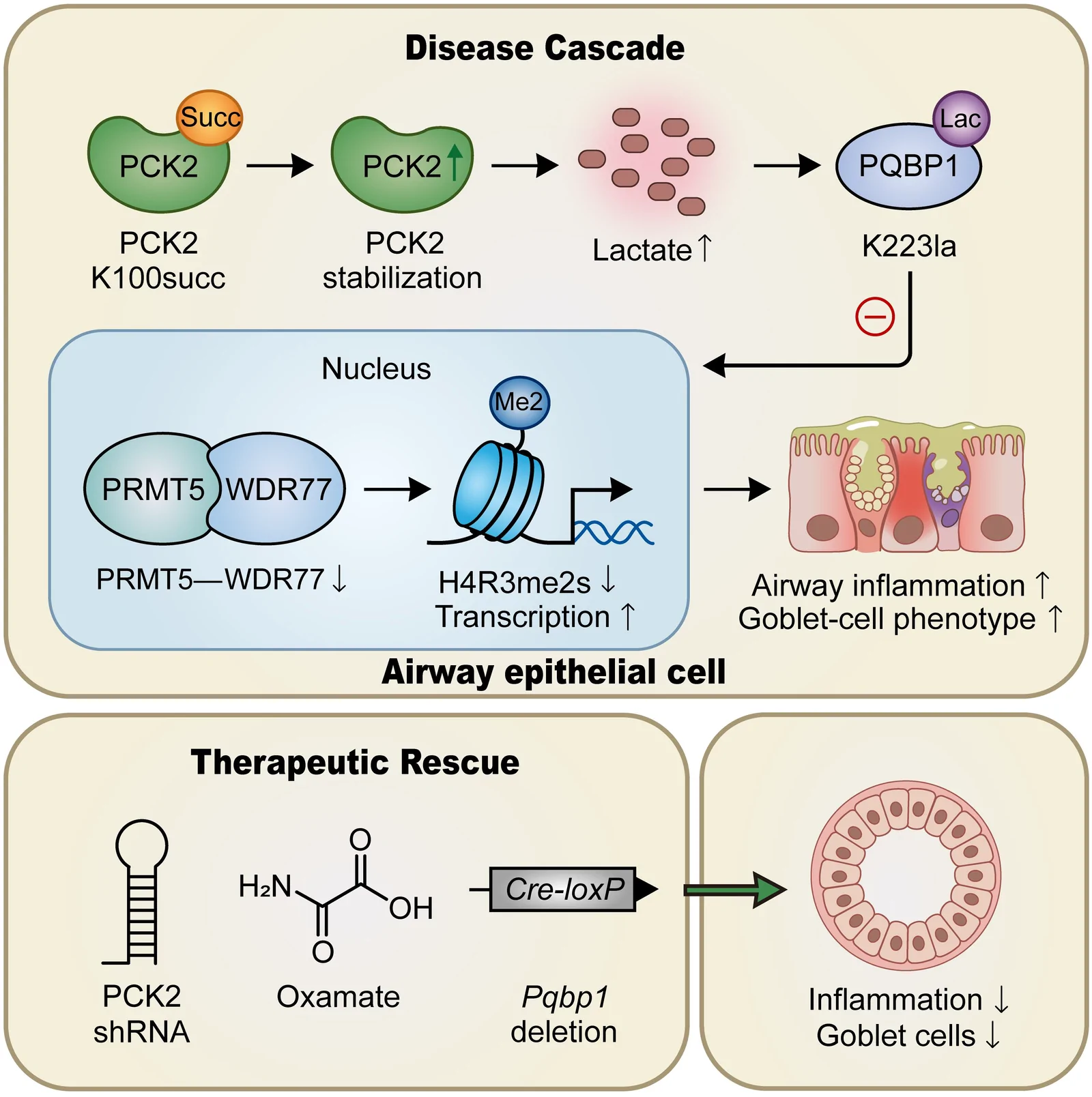
Preclinical PCK2–PQBP1–PRMT5 cascade and phenotypic rescue in airway epithelium. In HDM-induced asthma models, PCK2 K100 succinylation (‘K100succ’) stabilizes PCK2 and increases lactate, promoting PQBP1 K223 lactylation (‘K223la’). Lactylated PQBP1 inhibits PRMT5–WDR77, reduces H4R3me2s, and increases inflammatory transcription. The lower block summarizes PCK2 knockdown, oxamate treatment, and epithelial Pqbp1 deletion; each reduced airway inflammation and goblet-cell phenotypes in the tested models. The schematic label ‘Therapeutic Rescue’ denotes preclinical phenotypic rescue rather than a validated therapy. **Supplementary Figure S1** shows the extended map. Residue-specific validation in primary human asthmatic airway tissue remains required.

### 3.3 HDACs and sirtuins as context-dependent deacylases

HDACs and sirtuins oppose several lysine acyl modifications, but substrate specificity varies, and not all family members are established delactylases in airway cells. In an ovalbumin (OVA) model, eugenol reduced oxidative and inflammatory indices together with HDAC1 abundance and nuclear factor kappa B (NF-κB) p65 activity (49), but no lactylated substrate was identified.

Evidence for histone deacetylase (HDAC)-related control is similarly tiered. Jinwei decoction restored HDAC2-related glucocorticoid responsiveness through a miR-155-5p-associated pathway in COPD models (50), making its relevance to asthma indirect. chromosome 6 open reading frame 89 (C6orf89)-derived proteins provide a cell-biological precedent for compartment-specific HDAC regulation (19), but neither study identifies a lactylated airway substrate. The asthma-relevant question is which substrates are deacylated in each airway compartment, by which enzyme, and with what kinetics.

Sirtuin biology resists a simple protective-versus-pathogenic split. Icariside II activated a sirtuin 1 (SIRT1)-associated program and reduced NLRP3, transforming growth factor beta (TGF-β)/SMAD family member 3 (Smad3)/vascular endothelial growth factor (VEGF) signaling, alternatively activated (M2-like) macrophage polarization, and remodeling in chronic-asthma models (51). In contrast, IL-4-induced sirtuin 2 (SIRT2) promoted allergic inflammation, whereas the SIRT2 inhibitor AK-7 reduced NF-κB/MAPK signaling and enhanced NRF2-associated antioxidant responses (52, 53). These experiments define context-specific nodes, not fixed family-wide roles; AK-7 activity cannot be extrapolated to the SIRT2 inhibitor AGK2 or to SIRT2 blockade as a class effect.

Human samples and a neutrophilic-asthma model identify a macrophage SIRT6–LDHA–H4K12la circuit. SIRT6 deacetylates LDHA K261, increases lactate production, and drives H4K12la-dependent *Cxcl1*/*Cxcl2* transcription with subsequent neutrophil recruitment. Myeloid *Sirt6* deletion suppressed the program, and astragalin inhibited SIRT6 activity in mice with severe asthma (17). This pathway provides a direct mechanistic bridge from deacetylation of a metabolic enzyme to locus-specific histone lactylation. Because SIRT6 governs multiple homeostatic processes, translational work should begin with macrophage-restricted target engagement rather than systemic inhibition (**Figure 5**).

**Figure 5:**
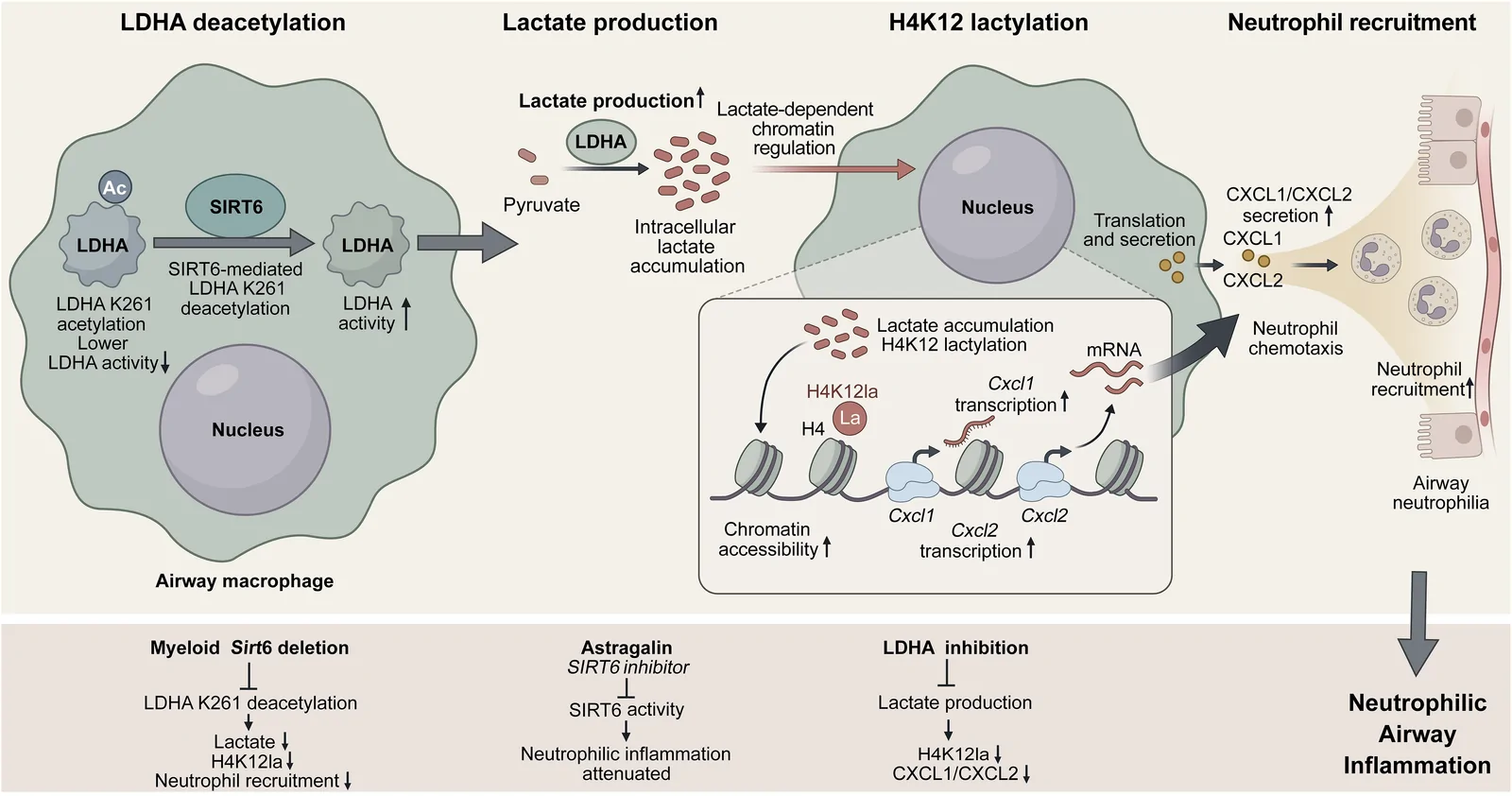
Macrophage SIRT6 couples LDHA deacetylation to H4K12 lactylation and neutrophil recruitment. SIRT6 deacetylates LDHA K261, increases LDHA activity and intracellular lactate production, and promotes H4K12la enrichment at the Cxcl1 and Cxcl2 loci. Increased CXCL1 and CXCL2 transcription and secretion drive neutrophil chemotaxis, airway neutrophilia, and neutrophilic airway inflammation. Myeloid Sirt6 deletion, astragalin-mediated SIRT6 inhibition, or LDHA-directed intervention attenuates this circuit. Evidence includes human airway samples and a neutrophilic-asthma model; independent residue- and locus-specific validation in human airway macrophages remains necessary.

### 3.4 BRD4 and super-enhancer organization

Asthma evidence places bromodomain-containing proteins 3 and 4 (BRD3/4) at the CXCL8 promoter in airway smooth-muscle cells and supports a role for BRD4 in acetylated-chromatin-dependent inflammatory transcription and remodeling (20, 48). It does not show that BRD4 reads lactylated chromatin. The histone H3 lysine 27 acetylation (H3K27ac)-defined interferon regulatory factor 4 (IRF4) super-enhancer that drives matrix metalloproteinase 12 (MMP12) was identified in experimental emphysema (54), whereas ZL0513 and ZL0516 show biochemical bromodomain 1 (BD1) selectivity and anti-inflammatory activity (55). These findings are adjacent chromatin precedents; direct recognition of a lactylated residue or reversal of an asthma-specific enhancer state remains to be demonstrated.

### 3.5 Dexamethasone and experimental resetting of the glycolysis–lactylation axis

In an OVA-induced murine model, dexamethasone lowered HIF-1α, glycolytic-enzyme expression, lactate abundance, protein lactylation, inflammation, pyroptosis-related signaling, and airway hyperresponsiveness (13). The coordinated reset is clear; causal ordering is not. Discriminating lactylation as mediator, metabolic recorder, or bystander will require site-specific rescue experiments rather than parallel measurement of declining markers (**Table 2**).

**Table 2:** Key cell-specific lactylation and adjacent chromatin pathways in asthma.

Pathway or regulator	Cell or disease context	Modification or target	Core consequence	Evidence tier and decisive gap	Ref.
Epithelial H3K18la–CSF1R	Airway epithelium	p300-associated H3K18la at the CSF1R promoter	CSF1R upregulation activates a CSF1–CSF1R–MAPK autocrine inflammatory circuit	Human samples, single-cell/ChIP-seq, and perturbation; endotype distribution not quantified	(15)
CD4^+^ T-cell H3K18la–DPP4	CD4^+^ T cells	H3K18la at the DPP4 promoter	DPP4 upregulation promotes Th17 differentiation and epithelial EMT/remodeling	Mouse and co-culture causality; airway-infiltrating patient T cells require locus-specific validation	(46)
PCK2–PQBP1–PRMT5 circuit	Airway epithelium	PCK2 K100 succinylation; PQBP1 K223 lactylation	PCK2 stabilization expands lactate; lactylated PQBP1 inhibits PRMT5–WDR77 and reduces H4R3me2s	Multi-omic and genetic mouse evidence; human residue-level detection absent	(16)
SIRT6–LDHA–H4K12la	Macrophages in neutrophilic asthma	LDHA K261 deacetylation; H4K12la at Cxcl1/Cxcl2	Increased lactate and CXCL1/2 drive neutrophil recruitment	Human samples plus myeloid perturbation; macrophage-restricted targeting must precede systemic inhibition	(17)
Dexamethasone-mediated metabolic reset	OVA asthma and macrophage systems	HIF-1α, glycolytic enzymes, lactate, and pan-Kla	Reduced lactate, protein lactylation, inflammation, and AHR	Mouse pharmacology; mediator-versus-bystander status of Kla requires rescue experiments	(13)
p300 writer and targetability	Molecular and asthma models	p300 catalytic domain	Candidate enzymatic link between lactate and histone lactylation; pharmacological tractability	Asthma H3K18la–CSF1R evidence is direct; docking/network studies lack intracellular engagement	(14, 18, 43, 44)
HDAC-associated regulation	Asthma, COPD, and cell models	HDAC1, HDAC2, and C6orf89-derived HDAC enhancers	Modulation of NF-κB signaling, steroid responsiveness, and compartmentalized deacylase activity	No airway site-specific delactylation demonstrated; COPD evidence is indirect	(19, 49, 50)
SIRT1/SIRT2 signaling	Experimental asthma	SIRT1-associated pathways; IL-4–SIRT2; AK-7	Context-dependent effects on inflammasome activity, remodeling, NF-κB/MAPK, and NRF2	Preclinical and compound-specific; substrates and inhibitor equivalence are unresolved	(51–53)
BET/BRD4 chromatin regulation	Asthma ASMCs and emphysema models	BRD3/4 at CXCL8; H3K27ac–IRF4 enhancer; BD1 inhibitors	Inflammatory transcription and remodeling	Strong acetylation precedent; direct recognition of lactylated asthma chromatin not shown	(48, 54, 55)
H3K14la–LRP5–β-catenin	COPD airway epithelium	LDHA-dependent H3K14la at the LRP5 promoter	β-Catenin activation and epithelial-barrier loss	Cross-disease mechanistic precedent, not an asthma pathway	(47)

## 4 Immunometabolic phenotypes of airway immune and structural cells

### 4.1 CD4^+^ T-cell subsets

Metabolic requirements differ among T-cell states. Activation and clonal expansion of T helper 2 (Th2) cells are supported by aerobic glycolysis and mechanistic target of rapamycin complex 1 (mTORC1), whereas regulatory T cells generally rely more heavily on fatty-acid oxidation and oxidative phosphorylation (OXPHOS) to maintain suppressive function (3). These are broad tendencies rather than fixed identities. After stimulation, proliferating CD4^+^ T cells from patients with asthma and from a mouse model of asthma produced more lactate than control cells. Dichloroacetate reduced lactate production, T-cell proliferation, IL-5, IL-17, and interferon-γ while increasing IL-10 and forkhead box P3 (Foxp3) in the experimental systems (56). The accompanying inverse association between serum lactate and forced expiratory volume in one second was observational and did not identify the cellular source of circulating lactate.

Two intervention studies expose separate control points in Th2 metabolism. Pterostilbene suppressed the GLUT1–mechanistic target of rapamycin (mTOR)–GATA-binding protein 3 (GATA3) axis, glycolysis, histone acetylation, and IL-4 production, attenuating HDM-induced disease in mice and reducing IL-4 release from patient-derived peripheral CD4^+^ T cells ex vivo (57). T-cell regulatory micropeptide (TREMP), encoded by the PPP2R5C long intergenic noncoding ribonucleic acid (lincR-PPP2R5C), localized to mitochondria and interacted with pyrroline-5-carboxylate reductase 1 (PYCR1) and sustained Th2 glycolysis and differentiation; deficiency of either component reduced allergic inflammation in mice, and both proteins were increased in Th2 cells from asthma samples (58). The first axis is pharmacological and partly human-ex vivo; the second is genetically resolved. Neither yet defines a chronic treatment strategy.

Glucose is only one substrate constraint on T-cell state. Tryptophan, glutamine, and arginine metabolism can influence differentiation through indoleamine 2,3-dioxygenase (IDO), the aryl hydrocarbon receptor, and mTOR (3). In a clinical study, high fractional exhaled nitric oxide (FeNO) identified a subgroup with increased epithelial inducible nitric oxide synthase and arginase 2, poorer lung function, and greater airway reactivity; epithelial overexpression linked this arginine-metabolic state to increased pyruvate-to-lactate flux (59). The data characterize a metabolic subgroup, but they do not reduce disease severity to a single arginine pathway.

Th9 and Th17 cells impose different metabolic demands. IL-4 and TGF-β drive Th9 differentiation through signal transducer and activator of transcription 5/6 (STAT5/STAT6)-linked programs (21), while PPARγ- and mTORC1-associated glycolysis supports Th9 function and may participate in an IL-9–MCT1 feedback loop. Evidence connecting that loop to non-eosinophilic hyperresponsiveness or corticosteroid response is still sparse. Th17-focused experiments are more mechanistically resolved: H3K18la enrichment at the DPP4 promoter increased DPP4 transcription and Th17 differentiation, whereas DPP4 inhibition or 2-deoxy-D-glucose suppressed the response and reduced epithelial EMT (46). Yet circulating T cells from severe allergic asthma showed downregulation, not uniform activation, of several metabolic pathways relative to mild disease (8). Sampling site, treatment exposure, and activation history are plausible explanations, but this discordance requires direct testing rather than *post hoc* harmonization.

### 4.2 ILC2 metabolic checkpoints

ILC2s coordinate glycolysis, mitochondrial metabolism, nutrient uptake, and organelle quality. In mouse and human ILC2 systems, pharmacological inhibition of glycolysis or HIF-1α reduced effector function, and conditional HIF-1α deletion attenuated IL-33- or HDM-induced airway inflammation in mice (60). The human component associates weaker HIF-1α/glycolytic activation with lower ILC2 activity; it does not test therapeutic HIF-1α inhibition.

Nutrient availability and inhibitory receptors add further checkpoints. Transferrin receptor 1 is rapidly induced during ILC2 activation, and restricting iron uptake reduces ILC2 expansion and effector function in murine and human cells. Iron deprivation paradoxically increases HIF-1α-dependent glycolysis while suppressing mitochondrial oxidation, and iron chelation or hypoferremia reduces airway hyperresponsiveness in mice (61). Systemic iron depletion is too biologically indiscriminate to infer a treatment strategy from this result. Prostaglandin E2 suppresses IL-33-dependent ILC2 activation through prostaglandin E2 receptor subtypes 2 and 4 (EP2/EP4)–cyclic adenosine monophosphate signaling and reduces STAT5 and MYC proto-oncogene transcription factor (MYC) programs and energy metabolism (62). Programmed cell death protein 1 (PD-1) imposes another brake: its absence shifts ILC2 metabolism toward glycolysis, glutaminolysis, and methionine catabolism, whereas a PD-1 agonist reduces airway hyperresponsiveness in a humanized mouse model (63).

Autophagy preserves a different form of metabolic balance. ILC2-specific loss of autophagy-related 5 impaired fatty-acid oxidation, increased glycolysis, and caused the accumulation of dysfunctional mitochondria, yet reduced type 2 cytokine production and airway hyperresponsiveness (64). Thus, increased glycolysis does not invariably indicate greater ILC2 function. BRG1-dependent chromatin states, NRF2-mediated redox adaptation, and melatonin-associated NADPH and glutathione production further influence ILC2 effector or memory programs in experimental allergic inflammation (29, 30).

### 4.3 Macrophage and dendritic-cell polarization

Asthma-specific macrophage and antigen-presenting-cell (APC) studies provide the primary evidence in this section. S100 calcium-binding proteins A8 and A9 (S100A8/A9) increased glycolysis, inflammatory polarization, and pyroptosis through Toll-like receptor 4 (TLR4)–myeloid differentiation primary response 88 (MyD88)–NF-κB signaling in OVA-exposed cells and mice; knockdown of either subunit reduced these responses, whereas S100A9 overexpression intensified them (69). Alternaria exposure altered PKM2 and HIF-1α in lung APCs, but both allosteric PKM2 activation with ML265 and PKM2 knockdown lowered glycolysis and APC activation in the reported model (70). Total PKM2 abundance cannot distinguish these states. Future biomarker studies should pair oligomeric or conformational measurements and catalytic flux with nuclear PKM2 and HIF-1α-associated signaling; drug studies should establish whether an intervention stabilizes catalytically active tetramers or suppresses noncanonical nuclear signaling before phenotypic outcomes are compared (70, 76, 91) (**Table 3**; **Supplementary Table S1**).

**Table 3:** Core experimental tests for causal metabolic–epigenetic mechanisms in asthma.

Priority question	Minimum design	Decisive readout	Go/no-go criterion	Failure guard	Ref.
Cell-resolved residue and locus causality	Sorted- or single-cell chromatin profiling plus site-specific loss-and-rescue	Cell-defined H3K18la, H4K12la, or PQBP1 K223 with matched target transcription	The modification precedes transcription and is reversed by site-specific rescue	Validated antibodies or proteomics are required; BRD4 occupancy is not Kla recognition	(15, 17, 45, 46, 48, 54)
Lactate source and compartment	¹³C tracing plus MCT perturbation and spatial or subcellular metabolomics	Production versus uptake; nuclear, cytosolic, and extracellular lactate; pH	Endogenous production and imported lactate are quantitatively separated	Bulk lactate, ECAR, or spatial proximity alone does not establish transfer	(15–17, 19, 26, 35–37, 40, 41, 93)
Writer/eraser and residue causality	Purified catalysis, catalytic-dead perturbation, site mutation, and residue-specific rescue	Site-specific Kla, enzyme activity, target transcription, and phenotype	Biochemical catalysis, cellular necessity, and residue-specific rescue converge	Enzyme perturbation may alter lactate indirectly; pan-Kla is insufficient	(14–17, 49–53)
PKM2 catalytic versus nuclear states	Oligomer/conformation assays, metabolic flux, and nuclear fractionation	Tetramer/dimer ratio, catalytic activity, nuclear PKM2, HIF-1α, and cytokines	Tetramer stabilization and noncanonical nuclear signaling are experimentally separated	Activation, tetramer stabilization, and depletion are not equivalent interventions	(70, 76, 91)
Small-molecule target engagement	SPR/ITC or enzyme assays plus CETSA or chemoproteomics in airway cells	Affinity, intracellular engagement, selectivity, exposure, and site-specific correction	Engagement occurs at pharmacologically relevant airway-cell exposure	Docking and model phenotypes cannot replace intracellular validation	(18, 44, 55)
Longitudinal human and delivery validation	Serial airway sampling linked to validated cell-selective aerosol platforms	Cell states, site-specific Kla, deposition, uptake, PK, target engagement, and outcomes	Temporal association replicates and delivery reaches the intended airway cell	Cross-sectional bulk signals and pan-Kla are insufficient; mucus penetration and clearance must be measured	(7, 8, 15–18, 46, 55, 93)

The extended design matrix is provided in **Supplementary Table S1**.

Outside asthma, β-catenin-driven mitochondrial reprogramming in infection models (65), endogenous α-ketoglutarate (αKG) flux in tumor microenvironments (66), exogenous αKG handling in dendritic cells (67), and NRF2-associated restoration of phagocytosis in COPD (68) show that myeloid metabolic states are plastic. These observations generate candidate mechanisms; they do not establish that the same αKG, β-catenin, or NRF2 circuits govern macrophage states in asthma.

### 4.4 Neutrophilic inflammation, inflammasomes, and pyroptosis

Neutrophils rely heavily on glycolysis. During severe infection-associated or neutrophilic airway inflammation, increased PKM2 activity, accumulation of succinate and lactate, and stabilization of HIF-1α can favor pro-IL-1β synthesis. Concurrent mitochondrial injury may release mtDNA, which can be sensed by absent in melanoma 2 (AIM2) and interact with NLRP3-containing inflammasome pathways. Caspase-1 cleavage of pro-IL-1β and gasdermin D (GSDMD) then enables IL-1β maturation and pore-forming cell death.

Direct asthma studies sharpen the inflammasome model. In induced sputum, macrophage Dectin-1 expression tracked with caspase-4 and neutrophil proportion. Dectin-1 activation increased caspase-11/4-dependent macrophage pyroptosis and CXCL1, CXCL3, CXCL5, and CXCR2 signaling in cells and mice, whereas caspase-11 inhibition suppressed the response (71). A prospective cohort of 48 patients with complementary models linked mixed eosinophilic–neutrophilic asthma to higher sputum NLRP3 and IL-1β and poorer lung function; perturbing NLRP3, GSDMD, or IL-1 signaling reduced epithelial pyroptosis or airway hyperresponsiveness (72). The pathway is biologically credible, but therapeutic efficacy and host-defense consequences have to be tested in patients rather than inferred from model suppression.

Substance P–neurokinin 1 receptor (NK1R) signaling and OIP5 antisense RNA 1 (OIP5-AS1)–Yes-associated protein (YAP) regulation provide two additional routes into pyroptotic injury. Clinical samples showed higher substance P and NK1R levels, while pathway inhibition reduced PI3K–AKT–NF-κB-dependent NLRP3 activation and epithelial pyroptosis in experimental systems (73). HDM-induced OIP5-AS1 stabilized YAP messenger RNA (mRNA) through eukaryotic initiation factor 4A3 (eIF4A3); OIP5-AS1 knockdown reduced NLRP3-related epithelial pyroptosis in cells and mice, and blood levels were higher in 32 patients (74). The unresolved competition is mechanistic rank: whether either branch dominates, cooperates with, or merely accompanies the Dectin-1 and IL-1 circuits across asthma phenotypes.

The selective NLRP3 inhibitor MCC950 reduced inflammasome activity and improved antibacterial responses in cystic-fibrosis models (22), information that is useful for host-defense assessment but secondary to asthma-specific perturbation. Treating AIM2–NLRP3–GSDMD as a single linear cascade would also overstate the current data. The evidence is better read as a set of convergent sensing and execution modules whose ordering may vary by cell type and inflammatory context.

## 5 Systemic metabolism, distal organ crosstalk, and tissue mechanics

To preserve the asthma-centered hierarchy, this section treats systemic and distal-organ findings as modifiers of airway disease rather than as evidence of airway lactylation. Human asthma observations are considered first where available, asthma models are used for mechanistic testing, and non-asthma or review-based mechanisms are retained only as hypotheses requiring airway-specific validation.

### 5.1 Gut–lung metabolic signaling

An OVA-induced mouse model provides a coherent gut–lung mechanism: live Leuconostoc mesenteroides MY2024 increased gut-derived cinnamic acid, activated colonic signal transducer and activator of transcription 1 (STAT1)–spermidine/spermine N1-acetyltransferase 1 (SAT1) signaling, accelerated spermidine catabolism, reduced systemic polyamine availability, and limited eukaryotic translation initiation factor 5A (eIF5A) hypusination in pulmonary macrophages (23). Alternative macrophage activation and allergic airway inflammation consequently declined. Human translation must first establish the direction and magnitude of cinnamic acid, spermidine, SAT1 activity, and eIF5A hypusination in the same individuals; long-term polyamine depletion cannot be assumed to be beneficial.

### 5.2 Obesity-related asthma and ILC2 plasticity

Obesity couples systemic metabolic inflammation to altered lung mechanics. A recent review proposes that visceral-adipose ILC2s become dysfunctional while pulmonary ILC2s acquire HIF-1α-associated glycolytic adaptation (75). Increased acetyl-CoA could reshape chromatin and promote ILC2 plasticity toward ILC1- or ILC3-like states, offering a model for non-type 2 inflammation. This is an attractive synthesis, not a traced lineage history. Direct lineage tracing is particularly necessary for the proposed transition toward neutrophil-associated programs and extracellular traps.

PKM2 dysregulation provides more direct experimental leverage in obesity-related asthma. HDM challenge induced glycolytic reprogramming in obese mice; the PKM2 tetramer stabilizer TEPP46 reduced airway inflammation without reversing remodeling, whereas epithelial *Pkm2* deletion reduced central airway resistance and inflammatory cytokines in both lean and obese allergic mice (76). Obesity also amplified allergen-induced epithelial dual oxidase 1 (DUOX1), IL-33, and type 2/type 17 responses in human nasal challenge and mouse experiments; leptin increased DUOX1-dependent IL-13 and mucin production in the murine arm (77). The separation between inflammatory improvement and persistent remodeling argues against treating PKM2 control as a single reversible disease switch. Short-term glucose intolerance without adiposity failed to reproduce the response, further separating obesity biology from hyperglycemia alone.

Circulating palmitate and dietary carbohydrate may add distinct metabolic pressures. Serum palmitic acid correlated with poorer lung function, higher blood neutrophil percentages, and greater corticosteroid requirements. In mice and epithelial cells, palmitate increased neutrophil chemokines and altered acylcarnitine/fatty-acid oxidation, sphingolipid, and arachidonic-acid pathways; CD36 scavenger receptor (CD36), Src family tyrosine kinase (Src), and extracellular signal-regulated kinase (ERK) were implicated in the mechanism (78). The clinical arm is correlational, whereas pathway perturbation is preclinical. In two adolescent cohorts, sucrose intake in males and starch intake in females were cross-sectionally associated with asthma prevalence. High-carbohydrate diets aggravated allergic inflammation, Th2/Th17 responses, oxidative stress, and glycolysis-related signaling in mice compared with a high-fat diet (79). Sex, energy intake, and correlated nutrients prevent a simple carbohydrate-to-asthma causal reading.

Multiomic profiling of obese children with allergic asthma identified higher leptin, lower plasma acetate, microbiome differences, higher fecal D-lactate and D/L-lactate ratios in poorer outcomes, and lower plasma citrate and dimethylsulfone in persistent asthma (80). The small observational dataset is valuable for mapping covariation across compartments; it is not a basis for diagnostic cutoffs.

### 5.3 Neuro–pulmonary effects of toxicant exposure

DiNP-exposed asthmatic mice provide one model of neuro–pulmonary toxicant injury (25). Brain tissue showed increased MDA and nitric oxide, decreased GSH and CAT, inhibition of respiratory-chain complexes I–IV, and markers of neuronal injury or apoptosis. The model demonstrates that allergic inflammation plus toxicant exposure can disrupt mitochondrial homeostasis beyond the lung. Its inferential boundary is equally clear: it does not establish neurodegeneration in patients with asthma.

Human data interrogate a different component of the lung–brain axis. Among 28 adults with asthma, individual cortisol responses to acute social stress were associated with salience-network glucose metabolism and with stronger allergen-provoked FeNO or IL-23A responses; greater chronic stress tracked with higher IL-17A expression after stress and allergen challenge (81). Acute stress did not uniformly increase airway inflammation. The signal is therefore one of interindividual neuroendocrine–metabolic coupling, not a universal causal effect of stress on exacerbation.

### 5.4 Cardiometabolic status and asthma prevalence

A cross-sectional analysis of 16,111 NHANES participants linked higher modified cardiometabolic index (MCMI) to self-reported asthma; the highest quartile had an adjusted odds ratio of 1.30 (82). MCMI directly indexes a systemic cardiometabolic phenotype, but the design leaves causal direction open to shared determinants, treatment, and behavior. Its current use is stratification, not mechanistic diagnosis.

### 5.5 Mechanical compression and club cell secretory protein 16-dependent metabolic homeostasis

Bronchoconstriction mechanically compresses the airway epithelium. Controlled compression reduced CC16 expression and altered enzymes in glycolysis, gluconeogenesis, and the pentose phosphate pathway (24); CC16-deficient epithelial cells showed lower basal oxygen consumption and diminished metabolic reserve. Mechanical stress may therefore narrow the epithelial response range during hypoxia or inflammation. The missing bridge is molecular: force sensing, cytoskeletal tension, nuclear deformation, CC16 transcription, and nuclear acylation have not yet been connected in one causal experiment.

Airway remodeling also intersects with proline metabolism. Plasma proline and pulmonary PYCR1 were increased in asthma samples and HDM-challenged mice. *Pycr1* deletion, pharmacological inhibition, or partial dietary proline restriction reduced EMT and remodeling in mice, accompanied by changes in mitochondrial fission, AKT–mTORC1, Wnt family member 3A (WNT3A)–β-catenin, and wider metabolic programs (83). A separate chronic-asthma study linked FOXK2 to SIRT2-dependent glycolysis and EMT; FOXK2 knockdown reduced collagen deposition and glycolysis in mice (84). Both pathways are experimentally coherent, but neither has yet supplied a time-resolved biomarker window for human remodeling.

### 5.6 Acute systemic hyperlactatemia is clinically distinct from tissue lactylation

Lactate measured during an acute exacerbation should not be interpreted as a direct readout of protein lactylation in the airway. In a prospective pediatric intensive-care cohort, 83% of children with acute severe asthma had hyperlactatemia, and 45% had lactate concentrations above 5 mmol/L; available lactate-to-pyruvate ratios indicated that most cases were type B rather than attributable to impaired oxygen delivery (85). A separate prospective study of 154 children found hyperlactatemia in 87% and lactic acidosis in 26% four hours after initial salbutamol treatment. Nebulized rather than inhaled salbutamol, age above six years, and hyperglycemia were associated with greater risk (86).

Adult studies show the same treatment-associated pattern. In 29 patients with acute severe asthma, lactate rose transiently during standardized therapy despite improved peak expiratory flow and blood-gas variables (87). In a prospective trial subanalysis, plasma albuterol concentration correlated with serum lactate after adjustment for severity, while hyperlactatemia did not predict poorer three-hour lung function, hospitalization, or one-week relapse (88). Compensatory tachypnea from metabolic acidosis can therefore masquerade as persistent bronchospasm. Only paired blood and tissue-resolved measurements can determine whether acute circulating lactate ever reaches concentrations or compartments that alter airway chromatin (**Table 4**).

**Table 4:** Major cell-specific immunometabolic programs and distal crosstalk in asthma.

Compartment	Dominant metabolic program	Key regulators	Asthma-relevant phenotype	Evidence tier and decisive gap	Ref.
CD4^+^ T cells and Th2 cells	High glycolytic flux and altered pyruvate handling	PDK; GLUT1–mTOR–GATA3; TREMP–PYCR1	T-cell expansion and IL-4/IL-5/IL-17/IFN-γ production	Patient-derived cells and mice; no chronic metabolic intervention in patients	(56–58)
Th17 cells	Promoter-specific histone lactylation	H3K18la–DPP4	Th17 differentiation and epithelial EMT	Direct experimental asthma mechanism; patient airway T-cell validation pending	(46)
Th9 cells	mTORC1/PPARγ-dependent glycolysis	STAT5/STAT6; IL-9–MCT1	Th9 differentiation and effector function	Plausible Th9 circuit; corticosteroid and non-eosinophilic phenotypes not prospectively linked	(21)
ILC2s	Coupled glycolysis/OXPHOS, nutrient uptake, autophagy, and redox control	HIF-1α, TfR1, EP2/EP4, PD-1, ATG5, NRF2, and BRG1	Cytokine output, memory-like states, and AHR	Strong preclinical evidence; direction changes with cell state and systemic context	(29, 30, 60–64)
Airway myeloid cells and APCs	Glycolysis, PKM2/HIF-1α signaling, pyroptosis, and H4K12la	PKM2; S100A8/A9–TLR4; SIRT6–LDHA; Dectin-1–caspase-4/11	Chemokine production, pyroptosis, and neutrophil recruitment	Asthma models plus selected human samples; catalytic and signaling nodes not yet clinically separable	(17, 69–71)
Airway epithelium	Glycolysis, inflammasome injury, and lipid/redox reprogramming	NLRP3–GSDMD–IL-1; NK1R–PI3K–AKT; OIP5-AS1–YAP; PKM2/leptin/DUOX1/palmitate	Barrier injury, mucus production, and mixed-granulocytic inflammation	Human associations and perturbation models; no single pathway accounts for mixed disease	(72–78)
Structural remodeling	Reduced metabolic reserve and proline-dependent remodeling	CC16 loss; PYCR1–AKT/mTORC1; WNT3A–β-catenin; FOXK2–SIRT2	Hypoxia susceptibility, EMT, collagen deposition, and remodeling	Experimental and asthma-sample evidence; temporal biomarker window not defined	(24, 83, 84)
Obesity and systemic metabolic stress	Metabolic inflexibility and adipose–lung crosstalk	HIF-1α, leptin, DUOX1, PKM2, and modified cardiometabolic index	Mixed type 2, type 17, and neutrophilic phenotypes	Review synthesis and cross-sectional data; adiposity, mechanics, and inflammation are non-equivalent	(75–78, 82)
Diet and gut–lung metabolism	Dietary metabolites, polyamine depletion, and altered lactate pools	STAT1–SAT1; spermidine–eIF5A; acetate, citrate, and D-lactate	Macrophage polarization and allergic inflammation	Mostly mouse or observational evidence; fecal D-lactate is not airway intracellular L-lactate	(23, 79, 80)
Neuroendocrine stress crosstalk	Cortisol and regional brain metabolic responses	Salience-network activity	Allergen-provoked FeNO, IL-17A, and IL-23A in selected individuals	Small challenge study; effect is interindividual rather than universal	(81)
Acute systemic hyperlactatemia	β_2_-agonist-driven glycolytic flux	Treatment-associated type B hyperlactatemia	Compensatory tachypnea despite bronchodilation	Clinical cohorts; circulating lactate cannot substitute for airway Kla	(85–88)
Non-asthma myeloid mechanisms	α-Ketoglutarate, serine, and mitochondrial reprogramming	β-Catenin; PHGDH–αKG–mTORC1; NRF2	Mechanistic hypotheses for immune-state regulation	Cross-disease precedent; asthma relevance requires direct perturbation	(65–68)

## 6 Therapeutic evidence and translational constraints

### 6.1 Biologics directed at IL-4Rα and IL-33

Dupilumab is a fully human monoclonal antibody against IL-4Rα, the shared receptor component for IL-4 and IL-13, and inhibits downstream Janus kinase–signal transducer and activator of transcription (JAK–STAT) signaling (2). Phase III trials established reductions in severe exacerbations, improvements in lung function, and decreases in type 2 biomarkers such as FeNO. These outcomes validate upstream cytokine blockade. Resetting glycolytic reprogramming or protein lactylation is a separate mechanistic claim and has not been measured directly in treated airway tissue.

Tozorakimab (MEDI3506) is a fully human anti-IL-33 antibody that blocks canonical suppression of tumorigenicity 2 (ST2) signaling and oxidation-dependent IL-33 signaling in experimental systems (89). The dual inhibitory mechanism is well defined. A metabolically or epigenetically stratified asthma trial, however, has not been performed.

### 6.2 Metabolic and inflammasome-directed interventions

Several interventions act upstream of lactate accumulation. Adhatoda vasica extract reduced airway inflammation and resistance in experimental severe asthma, restored prolyl hydroxylase domain-containing protein 2 (PHD2), promoted HIF-1α degradation, and improved mitochondrial morphology and bioenergetic indices in bronchial epithelial systems (90). 3PO and 3,6′-disinapoyl sucrose reduced inflammatory features through PFKFB3- or GALE-associated metabolic effects (33, 34), while Qufeng Xuanbi formula was linked to suppression of STING–HIF-1α signaling (31). Their common weakness is pharmacological definition: composition, target selectivity, tissue exposure, and human dose–response relationships are insufficiently resolved for the botanical preparations.

Mechanistically defined compounds help separate catalytic glycolysis from the moonlighting functions of glycolytic enzymes. TEPP46 stabilized tetrameric PKM2 and reduced IL-1β/STAT3-associated epithelial cytokines, eosinophilia, mucus metaplasia, and subepithelial collagen in HDM-challenged mice (91); in obese allergic mice, it reduced inflammation but left remodeling intact (76). Pterostilbene suppressed Th2 glycolysis and IL-4 (57), whereas 2-deoxy-D-glucose and HIF-1α inhibition curtailed ILC2 function (60). PFK15, epithelial HK2 knockdown, PCK2 short hairpin RNA (shRNA), and oxamate interrogated other points in the glycolysis–lactate network (16, 39, 40). Chronic-airway exposure, selectivity, and therapeutic windows have not been defined for any of these strategies in humans.

Molecular hydrogen shifted monocytes from patients and lungs from allergic mice away from the observed glycolytic pattern, restored indices of respiratory-chain and sirtuin function, and reduced murine inflammation (92). Because several metabolic variables moved together, the anti-inflammatory effect cannot be assigned to a single step. This ambiguity matters: indiscriminate glycolytic suppression could impair epithelial repair, lymphocyte memory, antimicrobial defense, or adaptation to hypoxia. Cell-selective delivery and pharmacodynamic readouts are therefore prerequisites, not refinements added after efficacy.

Asthma-specific experiments showed that MCC950 treatment or NLRP3 deletion reduced epithelial pyroptosis and airway hyperresponsiveness in mixed eosinophilic–neutrophilic models (72). MCC950 data from cystic-fibrosis models (22) are retained only to inform host-defense risk and do not strengthen efficacy claims in asthma. Lactate-transport evidence is even more indirect: in hyperglycemic, Pseudomonas-exposed cystic-fibrosis epithelium, MCT2-dependent lactate/proton secretion acidified airway-surface liquid and the MCT1/2 inhibitor AR-C155858 reduced the flux (93). This establishes a cystic-fibrosis transport mechanism, not evidence that MCT2 blockade will normalize asthma airway pH or reverse protein lactylation.

### 6.3 Chromatin-directed compounds and natural products

Asthma-specific perturbation provides the most relevant target evidence. p300 knockdown suppressed epithelial H3K18la and CSF1R-axis activity (15); astragalin inhibited SIRT6 and reduced neutrophil infiltration together with the LDHA–H4K12la program in neutrophilic-asthma models (17); and PCK2 knockdown or oxamate interrupted the PCK2–PQBP1–PRMT5 circuit and attenuated disease features in mice (16). These experiments support pathway dependence in the tested systems, but they do not establish intracellular target engagement, dose–response, or therapeutic efficacy in human airway tissue. Because p300, SIRT6, LDHA, and PRMT5 are central physiological regulators, development should proceed through airway- and cell-restricted target engagement rather than systemic pathway suppression.

Evidence for synthetic chromatin-directed candidates is lower tier. PU139 and PU141 were evaluated by docking, molecular dynamics, and binding-energy calculations; PU141 achieved a calculated binding free energy of −20.62 kcal/mol in the cited analysis (18). These in-silico studies prioritize a binding hypothesis but do not demonstrate biochemical inhibition or in-cell p300 engagement. ZL0513 and ZL0516 inhibit BRD4 BD1 with reported half-maximal inhibitory concentrations of 67–84 nM (55), yet intracellular engagement in human airway cells and correction of an asthma-relevant enhancer state remain unshown.

Natural products represent heterogeneous preclinical evidence. Eugenol reduced HDAC1/NF-κB activity in OVA-challenged mice (49), and icariside II activated a SIRT1-associated anti-inflammatory and antifibrotic program in chronic-asthma models (51). By contrast, Jinwei decoction was studied for HDAC2-related glucocorticoid responsiveness in COPD models (50), and Bailing Capsule constituents were linked to p300-related targets through network and experimental analyses (43). The first two are preclinical phenotypic findings, the third is cross-disease evidence, and the fourth does not resolve the active constituent or direct chromatin target. None currently demonstrates direct chromatin-target engagement in primary human asthmatic airway cells, and they should not be compared as one therapeutic class.

## 7 Critical mechanistic gaps, biomarker design, and experimental resolution

### 7.1 The metabolite-phenotype paradox

A metabolite is not intrinsically pro- or anti-inflammatory; its effect depends on source, concentration, subcellular location, transport route, and recipient cell. αKG illustrates the point. Endogenous αKG generated through the phosphoglycerate dehydrogenase (PHGDH)-linked serine pathway maintained an immunosuppressive macrophage phenotype in a tumor model (66), whereas exogenous αKG promoted tolerogenic properties in human monocyte-derived dendritic cells (67). The experiments differ in cell type, route, and microenvironment. They cannot be recast as bidirectional αKG action within one asthmatic niche.

The apparent lactate paradox likewise collapses when compartment and formulation are specified. MCT-dependent epithelial lactate/proton export acidified airway-surface liquid in a cystic-fibrosis model (93). A 1967 report, by contrast, described nonspecific improvement after intravenous molar sodium lactate in selected episodes of resistant acute asthma (94); the study predates contemporary trial standards and provides no mechanistic basis for efficacy. A further blind spot is intercellular flux: MCT4-mediated epithelial export may feed neighboring macrophages or simply dispose of excess lactate. Local concentration, systemic acid–base status, transporter identity, lactate salt, route, and timing must therefore be resolved before apparently contradictory observations are compared.

### 7.2 Subcellular compartmentalization and baseline transcriptional licensing

Subcellular placement can determine which acylation marks are written or removed. C6orf89-derived HDAC enhancers occupy different organelles (19), yet their airway substrates and kinetics have not been mapped. Baseline metabolic signaling can also license lymphocytes before overt activation. Tonic Ras guanyl-releasing protein 1 (RasGRP1)–mTORC1 signaling shapes oxidative-phosphorylation and spliceosomal translation in naïve T cells (95), and Raptor–mTORC1 is required for quiescence exit and Th2 differentiation (96). In T helper 1 (Th1) responses, autocrine cluster of differentiation 46 (CD46) cytoplasmic tail 1 (CYT-1) signaling increased L-type amino acid transporter 1 (LAT1) and GLUT1 expression (97). The CD46 study transfers a licensing concept, not a Th2-asthma mechanism.

Temporal ordering is the critical blind spot. Whether tonic nutrient transport and mTORC1 activity preconfigure histone lactylation or super-enhancer assembly in airway-infiltrating lymphocytes cannot be answered by comparing resting cells with terminally activated cells. Controlled activation time courses with matched flux, chromatin, and nascent-transcription measurements are required.

### 7.3 Redox adaptation and maintenance of a high-energy state

Some allergen-responsive effector cells maintain high glycolytic and oxidative capacity despite oxidative stress (11), contradicting a simple switch model in which glycolysis replaces OXPHOS. Compensatory mitochondrial biogenesis involving NRF2, mitochondrial transcription factor A, and microRNA-137 has been proposed as one explanation. The microRNA-137 evidence, however, comes from human neural stem cells (98). Importing that axis into asthma without airway-cell perturbation converts analogy into mechanism; the claim should remain explicitly hypothetical until tested in relevant cells.

### 7.4 Mechanotransduction and nuclear acylation

Mechanical compression reduces CC16 and epithelial metabolic reserve (24), but the force-to-chromatin sequence has not been resolved. Cytoskeletal tension, nuclear-pore deformation, metabolite access, and p300 or HDAC/sirtuin kinetics are plausible intermediates rather than established arrows. Nebulized ciglitazone offers an adjacent experimental clue: PPARγ activation reduced airway hyperresponsiveness, basement-membrane thickness, mucus, collagen, and TGF-β in OVA-challenged mice (99). It supports local PPARγ agonism as a remodeling strategy, but it does not close the mechanical-to-acylation gap.

### 7.5 Multi-omic and biophysical methods

Within the human asthma evidence summarized here, many measurements derive from mixed-cell sputum, biopsy, bronchoalveolar-lavage, or blood samples. Such specimens can support disease associations, but they cannot assign a lactylated residue to a defined cell or distinguish cell-intrinsic lactate production from uptake. Cell-type-specific multiomic datasets remain limited. Single-cell transcriptomic sequencing combined with cleavage under targets and tagmentation (CUT&Tag) or cleavage under targets and release using nuclease (CUT&RUN) can place BRD4, H3K27ac, or lactylation marks within defined cell states, while spatial metabolomics can map local metabolite availability (7, 15–17, 46). Longitudinal sampling can then test whether enhancer or lactylation changes precede cell-state transitions rather than merely accompanying them. Locus ChIP, global run-on sequencing, and enhancer-transcript measurements can further resolve glucocorticoid-receptor occupancy, KLF9-associated chromatin looping, and transcriptional timing (45, 54).

Small-molecule methods answer different layers of the same claim. Docking and molecular dynamics are triage tools; surface plasmon resonance, isothermal titration calorimetry, enzyme assays, structural analysis, cellular thermal shift assays, and chemoproteomics test affinity, catalysis, and intracellular engagement (18, 44). Spatial metabolomics paired with single-cell chromatin profiling can distinguish endogenous production from exogenous uptake of αKG or lactate. Controlled compression or stretch systems should then integrate metabolic flux with direct p300, HDAC, and sirtuin activity, rather than inferring mechanotransduction from endpoint chromatin marks.

Existing metabolomic studies expose the design problem. Bronchoalveolar lavage metabolomics in OVA-challenged mice detected higher lactate, malate, and creatinine amid broad carbohydrate and lipid changes; dexamethasone reversed many features but not those three metabolites (100). In obese children with allergic asthma, plasma and fecal metabolomics, inflammatory markers, and metagenomics resolved a multicomponent signature that no single analyte captured (80). Longitudinal human allergen challenge, in turn, identified asthma-specific epithelial glycolytic programs and pathogenic cell–cell circuits (7). None of these designs alone establishes metabolite-to-chromatin causality. Stable-isotope tracing, site-specific lactyl-proteomics, and perturbation must be combined within the same temporal experiment (**Table 3**).

### 7.6 Biomarker development

Biomarker development should be driven by a defined biological question rather than assay availability. A redox–metabolic–chromatin panel requires analytical validity, cell or compartment specificity, and prospective association with defined outcomes. 3-nitrotyrosine, 3-bromotyrosine, and 3-chlorotyrosine can be measured in bronchoalveolar lavage fluid or induced sputum (9), while nitric oxide or nitrite, MDA, GSH, CAT, and SOD provide peripheral redox readouts (49). Each captures oxidative biology; none specifically indexes airway lactylation.

Global pan-lysine lactylation (pan-Kla) should not be used as a stand-alone clinical biomarker. A bulk increase may reflect different cell proportions, modified residues, proteins, subcellular compartments, or lactate sources, and it cannot establish pathway causality. Biologically interpretable validation therefore requires a defined cell population, a residue-specific assay, a matched target or downstream readout, and evidence that the signal adds information beyond conventional inflammatory markers. Until these criteria are met, pan-Kla is a discovery-level readout rather than a diagnostic or endotype-defining measure (13, 15–17, 46).

Sputum lactate is closer to the airway compartment: levels were higher in asthma, especially in neutrophil-rich samples, correlated positively with IL-1β, and inversely with lung function (37). Site- and pathway-resolved assays could add epithelial H3K18la with CSF1R, macrophage SIRT6/LDHA activity with H4K12la and CXCL1/CXCL2, and PQBP1 K223 lactylation with PRMT5 activity (15–17). The immediate bottleneck is technical rather than conceptual: reproducible quantification in defined cells, followed by demonstration that the signal adds information beyond existing inflammatory markers.

Systemic and local markers should not be collapsed into one score. MCMI contributes a cardiometabolic dimension but derives from cross-sectional association (82). FeNO combined with epithelial inducible nitric oxide synthase and arginase 2 may resolve an arginine-metabolic subgroup (59). BRD4 abundance, PKM2 conformation, mtDNA fragments, mature IL-1β, and site-specific lactylation are mechanistic assays, whereas β_2_-agonist-associated blood lactate often reflects systemic type B hyperlactatemia (85, 88). A clinically useful panel must be prespecified against exacerbations, lung-function trajectories, and treatment response, with compartment-specific thresholds rather than pooled biomarker abundance.

## 8 A testable precision-endotype framework

### 8.1 Mechanistically anchored research profiles

Rather than forcing the reviewed pathways into a single metabolic endotype, the framework below separates five research profiles with different evidence depth and experimental burdens. They are organizing constructs, not diagnostic categories. Validation should be declared only through sequential analytical, biological, clinical, and external-replication gates.

Analytical qualification requires specific, reproducible assays in the intended cell and compartment. Biological support requires temporal ordering, pathway perturbation, and residue- or locus-specific rescue. Clinical replication requires longitudinal human-airway association and information beyond standard inflammatory or physiological markers; external validation requires independent cohort or laboratory replication under a prespecified analysis plan. Current evidence cannot justify one universal effect size, sample size, or replication count across these five profiles. Numerical criteria must instead be prespecified from pilot variance, endpoint, multiplicity, anticipated attrition, and intended use. Failure at any gate retains the construct as a research profile rather than a clinical endotype.

#### 8.1.1 Profile I

Type 2-high bioenergetic competence. NRF2-supported glycolysis and OXPHOS can sustain polyfunctional Th2 responses, while ILC2 activity depends on coordinated HIF-1α, nutrient uptake, redox control, and mitochondrial quality (11, 60–64). Th9-cell MCT1/IL-9 signaling may add a parallel glycolytic component (21). The CD46 CYT-1–LAT1/GLUT1 licensing circuit is conceptually relevant but derives from Th1 biology rather than asthma (97). A useful research panel would prioritize functional glycolytic and respiratory reserve, NRF2 activity, GSH/NADPH, MCT1, eosinophils, FeNO, and IgE over isolated transcript abundance. The profile earns clinical meaning only if this metabolic capacity predicts response beyond conventional type 2 biomarkers.

#### 8.1.2 Profile II

Neutrophilic glycolytic–lactylation–pyroptotic activity. The strongest candidate axis joins macrophage SIRT6-mediated LDHA K261 deacetylation to lactate production, H4K12la at CXCL1/CXCL2 loci, and neutrophil recruitment (17). Inflammasome activation, Dectin-1/caspase-4/11 signaling, mtDNA release, and epithelial or macrophage pyroptosis can converge on the same clinical phenotype (71, 72). They should not yet be drawn as one mandatory linear cascade. PKM2 is the principal mechanistic ambiguity: both allosteric activation and depletion reduced APC glycolysis in one model (70), implying that catalytic tetramerization and noncanonical nuclear signaling must be measured separately. Sputum neutrophils, PKM2 conformation, SIRT6/LDHA activity, H4K12la–CXCL1/2, mature IL-1β, GSDMD cleavage, Dectin-1/caspase-4, and mtDNA fragments would form a deliberately redundant discovery matrix. A composite endotype is justified only when the components co-segregate longitudinally and a single perturbation produces pathway-specific rescue.

#### 8.1.3 Profile III

Obesity- and mechanics-linked epithelial metabolic inflexibility. This profile is defined less by high glycolysis than by loss of reserve: elevated body mass index (BMI) or MCMI, reduced CC16, impaired epithelial respiration, and compression-sensitive metabolic remodeling (24, 75, 82). Leptin–DUOX1 signaling, palmitate exposure, PKM2 state, and ILC2 plasticity may stratify inflammatory pressure within that structural background (76–78). The key analytical separation is between adiposity, diet, systemic inflammation, and bronchoconstrictive mechanics; they can coexist but are not surrogate measures of one another. Longitudinal lung mechanics, CC16, respiratory reserve, leptin, palmitate, and DUOX1 should therefore be modeled as interacting variables rather than collapsed into a single obesity score.

#### 8.1.4 Profile IV

Gut–lung polyamine–hypusination signaling. The entire profile rests on one mechanistically coherent murine chain in which cinnamic acid activates STAT1–SAT1, accelerates spermidine catabolism, reduces macrophage eIF5A hypusination, and attenuates allergic inflammation (23). Human work should test that complete directionality in matched microbiome, plasma, and airway samples; isolated ‘low cinnamic acid’ or ‘low spermidine’ labels would misrepresent the model.

#### 8.1.5 Profile V

Compartmentalized lactylation and remodeling circuitry. At least three non-equivalent routes converge on structural airway injury. Epithelial p300-associated H3K18la activates CSF1R and a CSF1–CSF1R–MAPK autocrine loop (15). A separate epithelial PCK2 K100 succinylation–PQBP1 K223 lactylation circuit inhibits PRMT5–WDR77 and removes H4R3me2s-mediated repression (16). CD4^+^ T-cell H3K18la–DPP4 signaling supplies an extrinsic Th17 pressure that promotes epithelial EMT (46). The central paradox is coexistence: these histone and nonhistone acylation events may operate synchronously in a severe remodeling niche, define mutually exclusive trajectories, or emerge at different disease stages. Resolving that question requires a compartment-matched matrix of epithelial H3K18la/CSF1R, PCK2 K100 succinylation, PQBP1 K223 lactylation, PRMT5 activity, and T-cell DPP4 rather than a pan-Kla assay.

### 8.2 Staged validation gates and intervention logic

These profiles are designed to order validation and intervention experiments, not to justify multidrug regimens. The first test is anatomical: confirm the pathway in the relevant airway cell and compartment. The second is causal: demonstrate target engagement with genetics or one selective agent. Only then should upstream cytokine blockade be compared with interventions directed at glycolysis or lactate transport, lactylation writers or erasers, chromatin readers, and inflammasome execution (2, 15–18, 22, 33, 34, 55, 72, 89, 93).

Combination studies should begin only after reproducible monotherapy activity and molecular rescue have been shown. Factorial designs must distinguish additivity from redundancy and antagonism using standardized readouts of metabolism, inflammation, remodeling, host defense, pharmacokinetics, and toxicity. For p300, BRD4, sirtuins, LDHA, PCK2, and related central regulators, airway-restricted exposure is part of the experimental hypothesis because systemic inhibition can erase the very selectivity the endotype framework is meant to create (15–18, 55).

The near-term objective is therefore a measurable intervention point for each profile, not a preferred drug pair. Cross-model replication, residue- or locus-specific rescue, and confirmation in human airway samples set the entry criteria for biologic–metabolic or chromatin-directed combinations (**Table 5**).

**Table 5:** Mechanistically anchored asthma profiles and staged validation tests.

Research profile	Defining biology	Minimum evidence set	Experimental priority	Critical falsification test	Ref.
I. Type 2-high bioenergetic competence	NRF2- and mTORC1-supported glycolytic and oxidative capacity in Th2/ILC2 states	Functional glycolytic/respiratory reserve, NRF2, GSH/NADPH, GLUT1/MCT1, eosinophils, FeNO, and IgE	Test whether metabolic capacity predicts response beyond conventional type 2 biomarkers	The profile fails if functional metabolic readouts add no prospective information	(2, 11, 21, 33, 34, 43, 97)
II. Neutrophilic glycolytic–lactylation–pyroptotic activity	SIRT6–LDHA–H4K12la plus separable PKM2, mtDNA, and inflammasome modules	Sputum neutrophils, PKM2 conformation, SIRT6/LDHA, H4K12la–CXCL1/2, IL-1β, GSDMD, Dectin-1/caspase-4, and mtDNA	Separate catalytic PKM2, lactylation, and pyroptosis before testing IL-33, LDHA/MCT, NLRP3, or BRD4 interventions	The profile fails if the modules do not co-segregate or rescue independently	(17, 22, 55, 70–72, 89, 93)
III. Obesity/mechanics-linked epithelial metabolic inflexibility	Systemic metabolic pressure with reduced CC16 and epithelial respiratory reserve	BMI/waist, MCMI, leptin, palmitate, CC16, respiratory reserve, DUOX1, and airway mechanics	Model adiposity, diet, inflammation, and compression as interacting but non-equivalent exposures	The profile fails if loss of reserve is explained entirely by one exposure or treatment effect	(24, 31, 49, 50, 75–78, 82, 90, 99)
IV. Gut–lung polyamine–hypusination signaling	Cinnamic acid–STAT1/SAT1 signaling lowers spermidine and macrophage eIF5A hypusination	Microbiome function, cinnamic acid, polyamines, SAT1 activity, eIF5A hypusination, and macrophage state	Reproduce the full directional chain in matched human gut, plasma, and airway samples	The profile fails if isolated biomarkers do not preserve the murine pathway direction	(23, 51)
V. Compartmentalized lactylation and remodeling circuitry	Epithelial H3K18la–CSF1R, epithelial PCK2–PQBP1–PRMT5, and T-cell H3K18la–DPP4 routes	Cell-specific H3K18la/CSF1R, PCK2 K100, PQBP1 K223, PRMT5 activity, DPP4, and remodeling readouts	Determine whether the circuits coexist, alternate by stage, or define mutually exclusive trajectories	The profile fails if pan-Kla cannot be decomposed into residue- and cell-specific signals	(15–17, 46)
Cross-cutting: airway- and cell-restricted delivery	Local exposure is required when systemic inhibition disrupts central metabolic or chromatin functions	Regional deposition, diseased-mucus transport, cell-specific uptake, local/plasma PK, intracellular target engagement, pharmacodynamic response, and local/systemic toxicity	Map target-cell expression and airway-region trigger distributions before optimizing aerosol size, retention, mucus transport, uptake, and release kinetics	The platform fails if exposure misses the intended region or cell, lacks intracellular engagement, or cannot separate local activity from systemic exposure or airway injury	(15–18, 55, 93)

### 8.3 Airway-restricted delivery: technical gates and translational requirements

Systemic inhibition of central metabolic and chromatin regulators carries substantial risk of disrupting physiological functions outside the airway. Airway-restricted delivery may reduce collateral exposure, but local administration does not by itself establish regional or cellular selectivity. Candidate pH- or lactate-responsive aerosols, nanofibers, hydrogels, and engineered extracellular vesicles therefore remain platform concepts until the proposed trigger is mapped in the intended region of diseased human airways.

The first technical gate is transport through the asthmatic airway. Aerosol aerodynamic behavior and device compatibility must be linked to proximal and distal deposition, whereas disease-state mucus composition, viscoelasticity, mesh structure, and mucociliary clearance determine penetration depth, diffusion, residence, and loss. Formulation stability before and after nebulization, released dose, aggregation, and batch reproducibility should be measured under airway-relevant conditions; diffusion through healthy mucus surrogates cannot substitute for testing in diseased-airway material.

The second gate is cell-resolved pharmacokinetics and pharmacodynamics. Studies should quantify extracellular residence, uptake by the intended epithelial or immune cell, intracellular release or endosomal escape where applicable, target engagement, and residue- or locus-specific downstream response, with matched local-tissue and plasma time courses. A formulation should advance only if it reaches the intended airway region, crosses diseased mucus without unacceptable barrier injury, produces cell-selective engagement and pathway correction, preserves host defense, and maintains a local-to-systemic safety margin. Numerical go/no-go cutoffs should be prespecified from pilot assay variance, deposition, clearance, intended cell, and endpoint rather than treated as universal.

## 9 Conclusions: from pathway catalogues to testable profiles

Four asthma-associated lactylation circuits currently have the strongest direct mechanistic support across asthma-relevant systems: epithelial H3K18la–CSF1R signaling, PCK2-dependent PQBP1 K223 lactylation coupled to PRMT5 inhibition, CD4^+^ T-cell H3K18la–DPP4 control of Th17 differentiation, and macrophage SIRT6–LDHA–H4K12la induction of neutrophil chemokines. NRF2, HIF-1α, PKM2, and nutrient checkpoints shape the metabolic states that feed these circuits. The circuits should not be collapsed into a single ‘lactylation pathway’ or treated as validated clinical endotypes: their cell type, modified residue, transcriptional target, and evidence tier differ, and their prevalence, effect size, temporal stability, and distribution across human asthma phenotypes have not been established.

Metabolic regulation also governs Th2, Th9, ILC2, myeloid, and neutrophil function. Several detailed mechanisms in this review, however, come from COPD, cystic fibrosis, infection, emphysema, cancer, or neural models. Those studies are valuable when they define a testable precedent and misleading when they are imported as asthma mechanisms without perturbation. Gut metabolites, cardiometabolic status, and mechanical compression broaden the causal landscape, but they also create competing explanations that must be separated experimentally.

The next advance will come from temporal and spatial resolution rather than another static pathway inventory. Stable-isotope tracing should be paired with spatial metabolomics, single-cell chromatin profiling, nascent-transcription assays, site-specific lactyl-proteomics, and controlled mechanobiology in the same experimental sequence. Clinical studies should then test whether local lactate, defined lactylation sites, redox products, mtDNA, and systemic metabolic measures precede exacerbation, remodeling, or treatment response in prospectively specified cohorts.

Therapeutic development should follow the same evidentiary order. Biologics, metabolic inhibitors, chromatin-directed compounds, and natural products require airway target engagement, residue- or locus-specific pharmacodynamic readouts, additive benefit over existing therapy, and preservation of host defense and systemic metabolic homeostasis. For the five proposed profiles, progression from research construct to clinical endotype requires sequential analytical qualification, biological perturbation and rescue, longitudinal clinical replication beyond standard markers, and independent external validation under a prespecified analysis plan. Airway-directed interventions add further gates: regional deposition, transport through diseased mucus, cell-selective uptake, intracellular target engagement, matched local and systemic pharmacokinetics and pharmacodynamics, preservation of host defense, and a defined safety margin. Until those criteria are met, the profiles and intervention matrices should remain falsifiable experimental decision tools rather than diagnostic or therapeutic categories.

## Glossary

2-DG: 2-deoxy-D-glucose
3PO: 3-(3-pyridinyl)-1-(4-pyridinyl)-2-propen-1-one; a PFKFB3 inhibitor
Ac: acetyl group (figure symbol)
AGK2: sirtuin 2 inhibitor (compound designation)
AHR: airway hyperresponsiveness
AI: artificial intelligence
AIM2: absent in melanoma 2
AK-7: sirtuin 2 inhibitor (compound designation)
AKT: protein kinase B
αKG: alpha-ketoglutarate
AMP: adenosine monophosphate
APC: antigen-presenting cell
AR-C155858: monocarboxylate transporter 1/2 inhibitor (compound designation)
ASMC: airway smooth-muscle cell
ATG5: autophagy-related 5
BD1: bromodomain 1
BET: bromodomain and extra-terminal domain
BMI: body mass index
BRD3: bromodomain-containing protein 3
BRD4: bromodomain-containing protein 4
BRG1: Brahma-related gene 1
C6orf89: chromosome 6 open reading frame 89
CAT: catalase
CC16: club cell secretory protein 16
CD36: CD36 scavenger receptor
CD46: cluster of differentiation 46; membrane cofactor protein
CD4^+^: cluster of differentiation 4-positive
CETSA: cellular thermal shift assay
ChIP: chromatin immunoprecipitation
ChIP-seq: chromatin immunoprecipitation sequencing
COPD: chronic obstructive pulmonary disease
CSF1: colony-stimulating factor 1
CSF1R: colony-stimulating factor 1 receptor
CUT&RUN: cleavage under targets and release using nuclease
CUT&Tag: cleavage under targets and tagmentation
CXCL: C-X-C motif chemokine ligand (including CXCL1, CXCL2, CXCL3, CXCL5, CXCL8, and CXCL17)
CXCR: C-X-C motif chemokine receptor (including CXCR2 and CXCR4)
CYT-1: cytoplasmic tail 1 isoform of CD46
DiNP: diisononyl phthalate
DPP4: dipeptidyl peptidase 4
DUOX1: dual oxidase 1
ECAR: extracellular acidification rate
eIF5A: eukaryotic translation initiation factor 5A
EMT: epithelial–mesenchymal transition
EP2/EP4: prostaglandin E2 receptor subtypes 2 and 4
ERK: extracellular signal-regulated kinase
FeNO: fractional exhaled nitric oxide
Foxp3: forkhead box P3
FPR2: formyl peptide receptor 2
FUT8: fucosyltransferase 8
GALE: UDP-galactose 4-epimerase
GATA3: GATA-binding protein 3
GLRX1: glutaredoxin 1
GLUT1: glucose transporter 1
GSDMD: gasdermin D
GSH: reduced glutathione
H3K14la: histone H3 lysine 14 lactylation
H3K18la: histone H3 lysine 18 lactylation
H3K27ac: histone H3 lysine 27 acetylation
H4K12la: histone H4 lysine 12 lactylation
H4R3me2s: histone H4 arginine 3 symmetric dimethylation
HDAC: histone deacetylase (including HDAC1 and HDAC2)
HDM: house dust mite
HIF-1α: hypoxia-inducible factor 1 alpha
HK2: hexokinase 2
IDO: indoleamine 2,3-dioxygenase
IFN-γ: interferon gamma
IgE: immunoglobulin E
IKKϵ: inhibitor of nuclear factor kappa-B kinase subunit epsilon
IL: interleukin
IL-4Rα: interleukin-4 receptor alpha
ILC1/ILC2/ILC3: group 1, group 2, and group 3 innate lymphoid cells
IRF4: interferon regulatory factor 4
ITC: isothermal titration calorimetry
JAK: Janus kinase
Kla: lysine lactylation
KLF9: Krüppel-like factor 9
La: lactyl group or lactylation mark (figure symbol)
LAT1: L-type amino acid transporter 1
LDHA: lactate dehydrogenase A
lincR-PPP2R5C: long intergenic noncoding RNA transcript encoding TREMP
LRP5: low-density lipoprotein receptor-related protein 5
LTK14: p300-directed small molecule (compound designation)
M2: alternatively activated macrophage phenotype
MAPK: mitogen-activated protein kinase
MCC950: selective NLRP3 inhibitor (compound designation)
MCMI: modified cardiometabolic index
MCT: monocarboxylate transporter (including MCT1, MCT2, and MCT4)
MDA: malondialdehyde
MEDI3506: tozorakimab development code
MIF: macrophage migration inhibitory factor
miR/miRNA: microRNA
ML265: pyruvate kinase M2 activator (compound designation)
MMP12: matrix metalloproteinase 12
MPC2: mitochondrial pyruvate carrier 2
mRNA: messenger RNA
MS: mass spectrometry
mtDNA: mitochondrial DNA
mt-ND6: mitochondrially encoded NADH dehydrogenase 6
mTOR: mechanistic target of rapamycin
mTORC1: mechanistic target of rapamycin complex 1
mtROS: mitochondrial reactive oxygen species
MYC: MYC proto-oncogene transcription factor
MyD88: myeloid differentiation primary response 88
NADPH: reduced nicotinamide adenine dinucleotide phosphate
NAD^+^/NADH: oxidized/reduced nicotinamide adenine dinucleotide
NF-κB: nuclear factor kappa B
NK: natural killer
NK1R: neurokinin 1 receptor
NLRP3: NLR family pyrin domain-containing 3
NOX4: NADPH oxidase 4
NRF2: nuclear factor erythroid 2-related factor 2
OIP5-AS1: OIP5 antisense RNA 1
ORMDL3: ORMDL sphingolipid biosynthesis regulator 3
OVA: ovalbumin
OXPHOS: oxidative phosphorylation
p300: histone acetyltransferase p300 (EP300)
p65: nuclear factor kappa B p65 subunit (RELA)
pan-Kla: pan-lysine lactylation
PCK2: mitochondrial phosphoenolpyruvate carboxykinase 2
PD-1: programmed cell death protein 1
PDGF-BB: platelet-derived growth factor BB
PDK: pyruvate dehydrogenase kinase
PFK15: PFKFB3 inhibitor (compound designation)
PFKFB3: 6-phosphofructo-2-kinase/fructose-2,6-bisphosphatase 3
PHD2: prolyl hydroxylase domain-containing protein 2
PHGDH: phosphoglycerate dehydrogenase
PI3K: phosphoinositide 3-kinase
PK: pharmacokinetics
PKM2: pyruvate kinase M2
PPAR: peroxisome proliferator-activated receptor
PPARγ: peroxisome proliferator-activated receptor gamma
PPIF: peptidyl-prolyl cis-trans isomerase F (cyclophilin D)
PQBP1: polyglutamine-binding protein 1
PRMT5: protein arginine methyltransferase 5
PU139/PU141: p300-directed small molecules (compound designations)
PYCR1: pyrroline-5-carboxylate reductase 1
RNA: ribonucleic acid
ROS: reactive oxygen species
S100A8/A9: S100 calcium-binding proteins A8 and A9
SAT1: spermidine/spermine N1-acetyltransferase 1
scRNA-seq: single-cell RNA sequencing
shRNA: short hairpin RNA
SIRT: sirtuin (including SIRT1, SIRT2, and SIRT6)
Smad3: SMAD family member 3
SOD: superoxide dismutase
SPR: surface plasmon resonance
Src: Src family tyrosine kinase
ST2: suppression of tumorigenicity 2; interleukin-1 receptor-like 1
STAT1/3/5/6: signal transducer and activator of transcription 1, 3, 5, and 6
STING: stimulator of interferon genes
TEPP46: pyruvate kinase M2 activator and tetramer stabilizer (compound designation)
TfR1: transferrin receptor 1
TGF-β: transforming growth factor beta
Th1/Th2/Th9/Th17: T helper 1, 2, 9, and 17 cells
TLR4: Toll-like receptor 4
TREMP: T-cell regulatory micropeptide
TSLP: thymic stromal lymphopoietin
Ub: ubiquitin (figure symbol)
VDAC1: voltage-dependent anion channel 1
VEGF: vascular endothelial growth factor
VHL: von Hippel–Lindau tumor suppressor
WDR77: WD repeat domain 77
WNT3A: Wnt family member 3A
YAP: Yes-associated protein
ZL0513/ZL0516: BRD4 bromodomain 1 inhibitors (compound designations)
ZO-1: zonula occludens-1
