# Supplementary material for: Glycolytic reprogramming and cell-specific lactylation in asthma: an evidence-graded framework for testable metabolic-epigenetic profiles
Source: Front Immunol. 2026 Sep 15;17:1955426. doi: 10.3389/fimmu.2026.1955426 (PMC13621785; doi:10.3389/fimmu.2026.1955426)
Supplement: Supplementary file 1 [file Table1.docx]

**Supplementary Figure S1**

**Supplementary Figure S1. Extended PCK2–PQBP1–PRMT5 mechanism and perturbation map**

**Supplementary Figure S1. Full version of the original Figure 4. PCK2 K100 succinylation antagonizes ubiquitination-dependent proteasomal degradation, stabilizes PCK2, and expands intracellular lactate through metabolic rerouting.** Lactate-driven PQBP1 K223 lactylation inhibits PRMT5–WDR77, reduces H4R3me2s, and derepresses inflammatory and MAPK-related transcription. PCK2 shRNA, oxamate, and epithelial Pqbp1 deletion attenuated inflammation and goblet-cell phenotypes in asthma models. All intervention outcomes depicted are preclinical; residue-specific confirmation in primary human asthmatic airway tissue remains required.
