# Supplementary material for: Glycolytic reprogramming and cell-specific lactylation in asthma: an evidence-graded framework for testable metabolic-epigenetic profiles
Source: Front Immunol. 2026 Sep 15;17:1955426. doi: 10.3389/fimmu.2026.1955426 (PMC13621785; doi:10.3389/fimmu.2026.1955426)
Supplement: Supplementary file 2 [file Table2.docx]

**Supplementary Table S1**

**Supplementary Table S1. Extended experimental design matrix for causal metabolic–epigenetic mechanisms in asthma**

| **Priority question** | **Core approach** | **Essential readouts** | **Decision criterion** | **Interpretive failure mode** | **Ref.** |
| --- | --- | --- | --- | --- | --- |
| **Cell-resolved chromatin and locus causality** | scRNA-seq plus CUT&Tag/CUT&RUN, locus ChIP, and nascent-RNA assays | H3K18la, H4K12la, H3K27ac, BRD4, and target-gene transcription | Modification precedes transcription and is reversed by loss-and-rescue | Validate antibodies; BRD4 occupancy does not prove lactyl-lysine recognition | [15,17, 45–46,48,54] |
| **Nonhistone site function** | Quantitative lactyl/succinyl proteomics, site mutation, and interactomics | PCK2 K100, PQBP1 K223, ubiquitination, PRMT5–WDR77, and H4R3me2s | The residue is necessary and sufficient for the interaction and phenotype | Pan-Kla is insufficient; lysine mutants require biochemical validation | [16] |
| **Lactate source and metabolic flux** | ¹³C tracing, MCT perturbation, Seahorse analysis, and targeted metabolomics | Production/uptake rates, pyruvate fate, glycolytic reserve, and respiratory reserve | Cell-intrinsic production and imported lactate are quantitatively separated | Bulk lactate or ECAR alone cannot establish origin or mechanism | [15–17,26, 35,37,40–41,93] |
| **Spatial and subcellular compartmentalization** | Lactate/NAD⁺ sensors, fractionation, spatial metabolomics, and spatial transcriptomics | Nuclear, cytosolic, mitochondrial, and extracellular lactate; pH; NAD⁺/NADH | Tracing or transporter perturbation demonstrates metabolite transfer | Sensor calibration and fractionation artefacts must be controlled; proximity is not transfer | [16–17,19, 36–37] |
| **Writer/eraser assignment and pathway causality** | Catalytic-dead enzymes, knockout/knockdown, purified assays, and site-specific rescue | Site-specific Kla, enzyme activity, target transcription, and cellular phenotype | Purified catalysis, cellular necessity, and site-specific rescue converge | Enzyme perturbation may alter lactate indirectly rather than modify the site directly | [14–17, 49–53] |
| **PKM2 catalytic versus noncanonical states** | Native gels, size-exclusion chromatography, conformation assays, nuclear fractionation, and flux analysis | Tetramer/dimer ratio, catalytic activity, nuclear PKM2, HIF-1α, and cytokines | Catalytic and nuclear-signaling effects are experimentally separated | Activation, tetramer stabilization, and depletion are not equivalent interventions | [70,76,91] |
| **Small-molecule target engagement** | SPR/ITC, enzyme assays, CETSA or chemoproteomics, and structural analysis | Affinity, intracellular engagement, selectivity, exposure, and site-specific correction | Intracellular engagement occurs at pharmacologically relevant exposure | Docking and molecular dynamics cannot replace biochemical or cellular validation | [18,44,55] |
| **Longitudinal human-airway validation** | Serial allergen challenge with matched airway/blood sampling and multi-omics | Cell states, local lactate, Kla, cytokines, and clinical outcomes | Temporal order reproduces across airway and blood compartments | Treatment, sampling time, and compartment must be controlled; cross-sectional data are insufficient | [7–8,15–17,46] |
| **Mechanical-to-metabolic signaling** | Controlled compression/stretch with metabolic, nuclear-mechanics, and chromatin profiling | CC16, mitochondrial reserve, lactate flux, and p300/HDAC/SIRT activity | Force, metabolic intermediates, enzyme activity, and chromatin are linked sequentially | Direct force-to-lactylation arrows are premature without these intermediates | [24,99] |
| **Biomarker and delivery translation** | Targeted MS/validated assays plus cell-selective aerosol platforms | Analytical validity, deposition, uptake, PK, target engagement, and toxicity | A marker predicts biology or response and delivery reaches the intended cell | Pan-Kla is insufficient; responsive thresholds and cell specificity must be measured | [15–18,46, 55,93] |

Note. This table preserves the full design framework formerly presented as main-text Table 4. Numerical validation thresholds are not assigned because they require empirical prespecification for the intended assay, endpoint, and cohort.
